# Sex-Specific Changes in Cardiac Function and Electrophysiology During Progression of Adenine-Induced Chronic Kidney Disease in Mice

**DOI:** 10.3390/jcdd11110362

**Published:** 2024-11-07

**Authors:** Valentina Dargam, Anet Sanchez, Aashiya Kolengaden, Yency Perez, Rebekah Arias, Ana M. Valentin Cabrera, Daniel Chaparro, Christopher Tarafa, Alexandra Coba, Nathan Yapaolo, Perony da Silva Nogueira, Emily A. Todd, Monique M. Williams, Lina A. Shehadeh, Joshua D. Hutcheson

**Affiliations:** 1 Department of Biomedical Engineering, Florida International University, Miami, FL 33174, USA; vdargam@fiu.edu (V.D.);; 2 Department of Biological Science, Florida International University, Miami, FL 33199, USA; 3 Department of Medical Education, Leonard M. Miller School of Medicine, University of Miami, Miami, FL 33136, USA; 4 Department of Medicine, Division of Cardiology, Leonard M. Miller School of Medicine, University of Miami, Miami, FL 33136, USA; 5 Biomolecular Sciences Institute, Florida International University, Miami, FL 33199, USA

**Keywords:** chronic kidney disease, heart disease, echocardiography, ECG, sex differences

## Abstract

Chronic kidney disease (CKD) and cardiovascular disease (CVD) often co-exist, with notable sex-dependent differences in manifestation and progression despite both sexes sharing similar risk factors. Identifying sex-specific diagnostic markers in CKD-induced CVD could elucidate why the development and progression of these diseases differ by sex. Adult, C57BL/6J male and female mice were fed a high-adenine diet for 12 weeks to induce CKD, while control mice were given a normal diet. Adenine-treated males showed more severe CKD than females. Cardiac physiology was evaluated using electrocardiogram (ECG) and echocardiogram markers. Only adenine-treated male mice showed markers of left ventricular (LV) hypertrophy. Adenine males showed markers of LV systolic and diastolic dysfunction throughout regimen duration, worsening as the disease progressed. Adenine males had prolonged QTc interval compared to adenine females and control males. We identified a new ECG marker, S_peak_-J duration, which increased with disease progression and appeared earlier in adenine-treated males than in females. We identified sex-dependent differences in cardiac structure, function, and electrophysiology in a CKD-induced CVD mouse model, with adenine-treated males displaying markers of LV hypertrophy, dysfunction, and electrophysiological changes. This study demonstrates the feasibility of using this model to investigate sex-dependent cardiac differences resulting from CKD.

## 1. Introduction

Chronic kidney disease (CKD) is associated with notable sex-dependent differences in cardiovascular disease (CVD). A recent study of adults with CKD found that women, when compared to men, have a lower incidence of atherosclerotic events, heart failure (HF), CVD mortality, and mortality from non-CVD causes [1]. However, women present with CKD more frequently than men [2] and, at end-stage CKD, experience higher rates of cardiovascular-related events compared to men [3]. These sex-dependent differences in the risk of CVD exist despite men and women having similar incidences of traditional risk factors, including obesity and hypertension [4]. Understanding why the development and progression of CVD in CKD patients differ by sex, despite sharing similar risk factors, is crucial for developing targeted strategies to prevent and manage CVD in CKD patients.

It is particularly important to identify and monitor patients with both CKD and CVD since the co-occurrence of both diseases is associated with a higher rate of poor outcomes [5]. An electrocardiogram (ECG) is a noninvasive and rapid screening test that can identify abnormalities in cardiac function. ECG-specific markers can help identify the risk of HF and associated adverse events [6]. However, common ECG markers of CVD are often absent in CKD patients, which can negatively impact disease identification and monitoring [7]. A review conducted in 2019 by Skampardoni et al. determined that “conventional ECG markers are not reliable for risk stratification in the renal populations” [8]. To complicate matters, sex differences in ECGs among the general population are well-known—with females exhibiting a higher heart rate (HR), shorter PR interval and QRS duration, and longer corrected QT duration [9]. Reliable and predictive sex-specific ECG markers of CKD-induced CVD are needed to identify patients requiring further diagnostics tests, management, and intervention.

The adenine-induced CKD mouse model, which involves feeding mice a high-adenine diet for several weeks, is commonly used to study CKD without invasive surgical procedures [10,11,12]. An adenine regimen induces renal failure by causing the accumulation of toxic 2,8-dihydroxyadenine crystals in the kidneys, which obstruct the renal tubules, leading to inflammation, tubular damage, and fibrosis [11,13]. Over time, this results in progressive kidney damage and impaired renal function, mimicking CKD. We previously showed cardiac structural and functional changes in adenine-induced CKD mice, with male mice exhibiting an increase in left ventricular (LV) ejection fraction, stroke volume index, and mass compared to control [14]. However, female mice were not included in the study. To our knowledge, no reports exist on potential differences in cardiac morphology and function between male and female mouse models of CKD. Studies on humans have focused on sex differences in CVD outcomes within the CKD population [15,16], and sex-dependent changes in cardiac structure and function in CKD-induced CVD have not been well studied.

In this study, we assess cardiac structural and functional changes during the progression of CKD-induced CVD using electrocardiography and echocardiography to determine whether these changes differ based on sex.

## 2. Materials and Methods

**Figure 1:**
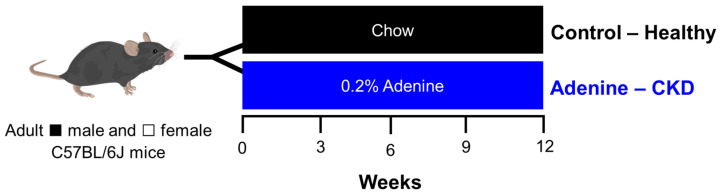
*Experimental design.* Adult (8–10 weeks of age) C57B/6J ■ male and □ female mice were either fed a normal chow or high adenine diet to serve as healthy Controls or to induce CKD, respectively. Electrocardiogram signals, echocardiograms, blood, and tissues were collected or analyzed every three weeks, starting from week 3 of the regimen.

*Mouse Model.* Mice of C57BL/6J background were bred and housed on a 12:12 h light/dark cycle at room temperature (20–26 °C). Adult mice, aged 8–10 weeks old and of both sexes, were assigned to either the Control group, representing healthy controls, or the Adenine group, which developed CKD and cardiac dysfunction (Figure 1). Mice in the Control group were fed a normal chow diet (5V75—PicoLab^®^ Verified 75 IF, TestDiet^®^). Mice in the Adenine group were fed a high-adenine diet (0.2%), developed by Tani et al. to induce CKD [10]. The high adenine diet was custom-made by increasing adenine (Adenine, 99%, Thermo Scientific Chemicals, Ward Hill, MA, USA) levels of the normal chow diet to 0.2%. Mice were sacrificed at 3, 6, 9, and 12 weeks after induction of CKD, with age-matched controls sacrificed at the same time points (Figure 1). Echocardiogram and ECG signals were recorded at the same time points as described above. At experiment termination, body weight was assessed, and hearts were excised and weighed. A sample size of 8–10 mice was used for each time point, group, and sex.

*Measurement of Urea Nitrogen.* Kidney function was assessed throughout disease development by measuring blood urea nitrogen (BUN) levels using the Urea Nitrogen (BUN) Colorimetric Detection Kit (Catalog Number EIABUN, Thermo Fisher Scientific, Waltham, MA, USA). Plasma samples were collected by immediately adding the appropriate volume of 0.5 M ethylenediaminetetraacetic acid solution to each blood sample, ensuring thorough mixing to achieve a final concentration of 4 nM. The assay was performed following the manufacturer’s instructions, with each sample diluted at a 1:20 ratio using ultrapure water. Two technical replicates were used per biological replicate (i.e., per mouse).

*Echocardiography.* Cardiac hemodynamic and structural parameters were evaluated using a high-frequency ultrasound imaging system (Vevo F2, FUJIFILM VisualSonics, Toronto, ON, Canada) as previously described [14]. In short, we anesthetized the mice by inhalation with an isoflurane and oxygen mixture, body temperature was maintained using a heated platform and lamp, and their physiological state (HR and respiratory rate) was monitored by recording ECG signals using subcutaneous electrodes. Cardiac parameters of structure and function were obtained using B-mode, M-mode, Pulse-Wave Doppler, and Pulse-Wave Tissue Doppler ultrasound modalities. Cardiac parameters were analyzed to evaluate overall cardiac function, systolic and diastolic function, and LV wall thickness. For measures of volume and wall size, which include ejection fraction and wall thickness, parameters were obtained in parasternal short-axis view using M-mode images. Diastolic dysfunction was assessed in apical four-chamber view, using pulse-wave Doppler to quantify mitral valve blood flow parameters and pulse-wave tissue Doppler to quantify mitral annular displacement. The echocardiogram data were analyzed and quantified with Vevo LAB software (Version 5.8.2, FUJIFILM VisualSonics, Toronto, ON, Canada). All measurements per mouse were obtained from at least 3 cardiac cycles and the average value was used as the biological replicate for group comparison. A sample size of 6–8 mice was used for each time point, group, and sex.

*Electrocardiography.* Cardiac electrical signals were recorded using Lab Rat Ephys system (Tucker-Davis Technology, Alachua, FL, USA) and needle electrodes (29-gauge, AD Instruments, Sydney, Australia). Mice were anesthetized with an inhalant anesthetic administered using a precision vaporizer. Mice were first placed in an induction chamber with isoflurane levels of 2–4% *v*/*v* and an oxygen flow rate of 1–2 L per minute. Once anesthetized, isoflurane levels of 0.5–2.5% *v*/*v* with oxygen flow rates of 1–2 L per minute were maintained with a facemask. The ratio of oxygen to isoflurane in the inhalant anesthetic was adjusted to maintain a respiratory rate between 60 and 80 breaths per minute, minimizing respiratory artifacts in the ECG signals caused by deep anesthesia. Four needle electrodes were placed in the limbs subcutaneously (channel 1 to right forelimb, channel 2 to left forelimb, channel 3 to left hindlimb, and channel 4 to ground right hindlimb). ECG signals were recorded using SynapseLite (Tucker-Davis Technology) for approximately 60 s at a sampling frequency of 12,207 Hertz. Lead I was used for quantification of ECG parameters. A sample size of 8–10 mice was used for each time point, group, and sex. All cycles found in the recording window were used for analysis, with a minimum of 88 cycles per recording averaged to obtain each biological replicate. The location of each ECG marker was obtained from the average ECG signal.

ECG signals were processed with MATLAB (2023b, MathWorks Inc., Natick, MA, USA) using a Gaussian filter over a five-element sliding window and a passband filter (5–500 Hz) to remove high-frequency electromyographic noise. It is important to note that mouse ECG signals have different characteristics from human ECG signals [17]. For example, the QRS complex is not a good measure of total ventricular activation time in mice [17]. Therefore, markers specific to murine ECG signals were identified for this study. A custom algorithm was used to find the location of the following ECG markers: P_start_, P_peak_, P_end_, Q_start_, Q_peak_, R, S_peak_, S_end_, J, p, T_peak_, and T_end_. For each recording, we used an R-wave amplitude threshold to identify the location of each R-wave peak, which is indicative of a new cardiac cycle. These locations were then used to find the RR interval duration. To find the remaining waves, the location of all local maxima/minima values and zero-crossings between each R-to-R segment were identified. The p marker was measured from the peak negative deflection of the Q wave to the offset of the J wave. The offset of the J wave, or p, was defined as the beginning of negative values following the local maximum of the J wave. For mice with a J wave below zero, the S_end_ and *p* value were undefined, and these values were excluded from the analysis. For each recording, the location of each ECG marker was blindly verified to ensure the algorithm was able to accurately detect each ECG marker. In the cases where the algorithm failed to accurately detect the ECG marker, we manually selected the location.

In both mice and humans, the most common ECG parameter durations reported in the literature are the following: RR interval, PR interval, P-wave duration, QRS interval, ST interval, and QT interval [18,19,20,21,22,23]. In this study, we report both traditional ECG parameters and the duration of additional, non-traditional markers in an effort to identify new ECG parameters that can detect CKD-induced cardiac abnormalities. The duration of all ECG segments (region between the end of one wave to the start of another) and intervals (region between the start of one wave to the end of another) were identified.

In humans, the duration of ECG intervals, such as the QT interval, is corrected to account for HR differences [24]. We previously found that mice on the Adenine diet have a lower HR [14], which corresponds to a longer cardiac cycle duration. Speerschneider et al. showed that the QT interval duration in anesthetized C57BL/6J mice does not correlate well with HR and therefore should not be adjusted [21]. However, Boukens et al. argued that HR dependence could manifest in certain disease models, and ECG parameters should be corrected if a correlation exists [17]. To determine whether HR influences ECG parameters and ensure that the changes observed are not due to HR differences, we assessed whether a linear correlation exists between the duration of ECG characteristics and cycle duration (RR interval duration). To determine this, a linear regression model was fitted to RR interval duration and each ECG parameter, with the data separated by regimen type and combined for both groups. If the absolute value of the correlation coefficient was greater than 0.70, a strong linear relation to cycle duration was assumed and the parameter was corrected to account for HR (i.e., cycle duration). For the corrected parameter, the following formula was used to remove the linear effect of HR: Corrected Parameter = Parameter − Slope × [RR Interval duration − Mean RR Interval Duration]. The slope was extracted from the fitted linear model, and the mean RR Interval Duration was calculated using all sexes, regimen types, and time points.

*Tissue Collection and Morphometry.* Hearts were flushed with 1× PBS by puncturing the right ventricle. Heart chambers were resected and weighted to determine muscularization. Some hearts from mice at week 12 of the regimen were set aside for histological examination of chamber size using hematoxylin and eosin stain. Those hearts were fixed in 10% neutral buffered formalin for 24 to 48 h and then embedded in paraffin. Transverse 5 μm sections of the ventricles were stained with hematoxylin and eosin according to standard histological procedures and 2× images were obtained using a BZ-X810 microscope (Keyence Corporation, Itasca, IL, USA).

*Statistical Procedures.* All parameters are presented as the mean ± standard deviation of the mean. The total number of mice per group, per time point, for each parameter is represented by *n*, separated by sex into females (□ F) and males (■ M). The statistical significance of differences at each timepoint, considering sex (male or female) and regimen type (Control-Chow or CKD-Adenine), was assessed using a two-way ANOVA with Bonferroni correction. A one-way ANOVA with Bonferroni correction was used to detect significance due to disease progression for each sex and its corresponding regimen type. A *p*-value of ≤0.05 was considered statistically significant.

## 3. Results

### 3.1. Sexual Dimorphism in the Progression of Adenine-Induced CKD

**Figure 2:**
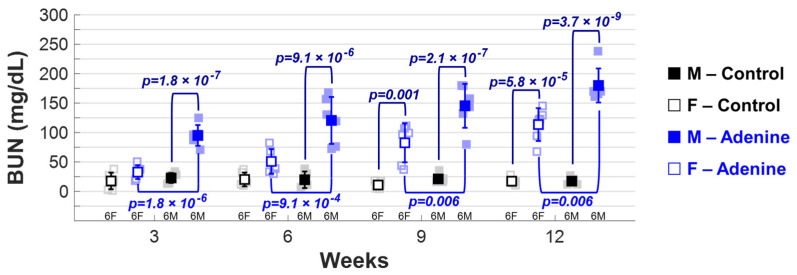
*Progression of CKD in adenine-fed mouse model.* Levels of blood urea nitrogen (BUN) throughout regimen duration. The statistical significance of differences at each timepoint, considering sex (■ male and □ female) and regimen type (Control–Chow or CKD–Adenine), was assessed using two-way ANOVA (Bonferroni) and shown with a navy-blue *p*-value. If a sex-dependent interaction is present at any time point, it is shown with black *p*-values for the Control group and blue *p*-values for the Adenine group.

BUN levels were assessed to determine whether the progression of CKD varied due to sex. Adenine males had significantly higher BUN levels than Control males and Adenine females at all timepoints (Figure 2). Adenine females exhibited significantly higher BUN levels starting at week 6 of the diet compared to time-matched Control females (Figure 2). At weeks 3, 6, 9, and 12, BUN levels in Adenine-treated males were 2.91, 2.37, 1.76, and 1.59 times higher than those in Adenine-treated females.

### 3.2. Body Weight Affected by Regimen Type, Duration, and Sex

**Figure 3:**
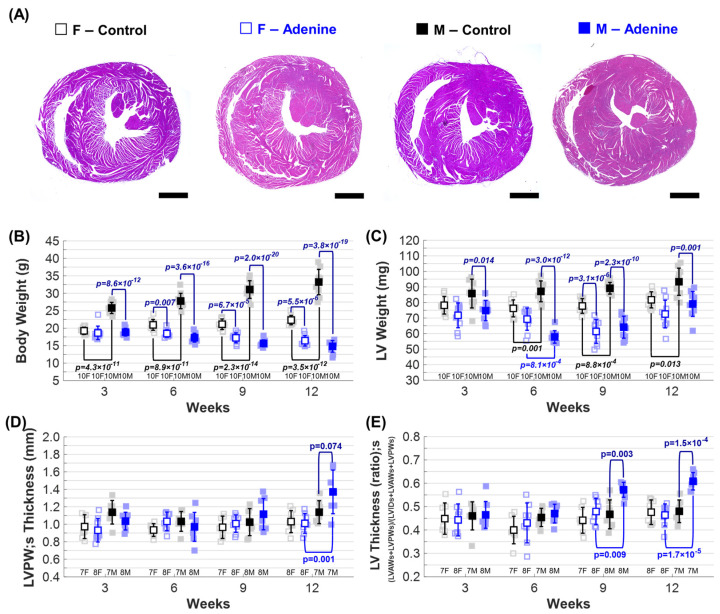
*Morphological and echocardiographic evaluation of left ventricular (LV) remodeling*. Changes in ■ male and □ female mice throughout the progression of either **Control** (**Healthy**) or **Adenine** (**CKD**) regimen: (**A**) Representative images of cardiac tissues using hematoxylin & eosin (H&E) after 12 weeks of either dietary regimen showing size of left and right ventricles (scale bar 50 µm). Sex-dependent differences based on regimen type for the following: (**B**) body weight, (**C**) left ventricular (LV) weight, (**D**) left ventricular posterior wall (LVPW), and (**E**) LV thickness. Measures of LV wall thickness and inner diameter were calculated at systole (s). Results are presented as mean ± standard deviation. The statistical significance of differences at each timepoint, considering sex (■ male and □ female) and regimen type (Control–Chow or CKD–Adenine), was assessed using two-way ANOVA (Bonferroni) and shown with a navy-blue *p*-value. If a sex-dependent interaction is present at any time point, it is shown with black *p*-values for the Control group and blue *p*-values for the Adenine group.

For both Control and Adenine groups, there were statistically significant differences in body weight throughout regimen duration due to regimen type and sex. For the Control group, male mice were significantly heavier than female mice at all timepoints (Figure 3B) and both male and female mice had an increase in body weight as a function of time (Supplementary Figure S1A). A sex-dependent difference was observed between the male and female mice in the Control group, with male mice having a higher body weight than females at each time point (Figure 3B). When compared to the Control group, the body weight of both male and female mice in the Adenine group was significantly decreased throughout disease progression (Supplementary Figure S1A). Male mice in the Adenine group had significantly lower body weight than age-matched Controls, with the Adenine-fed males being 44.55% lighter than their age-matched Control at week 12. Adenine-fed females were 73.57% lighter than age-matched Controls at week 12. For both sexes in the Adenine group, body weight decreases throughout disease progression (Supplementary Figure S1A).

### 3.3. Decline in Body Weight Affects Normalization of Heart Chambers in CKD Mouse Model

The weight of heart chambers in rodents is usually normalized to either body weight or tibia length to account for variations in body size [25]. Body weight normalization allows for a more accurate comparison of heart and chamber size relative to the overall size of the animal, providing a standardized way to assess cardiac hypertrophy. We verified the correlation between heart chamber size and body weight, heart weight, and tibia length to determine if the significant weight loss in the Adenine group and sex affected either normalization method (Supplementary Table S1). Heart weight and LV weight showed a linear relationship to body weight in the Control group (R = 0.6609 and 0.7062, respectively). On the contrary, heart weight and LV weight correlated poorly with body weight in the Adenine group (R = 0.2051 and 0.1049, respectively). Both groups showed better linear correlations when normalizing chamber size to heart weight, with LV weight exhibiting a linear relationship in both the Control and Adenine groups (R = 0.8282 and 0.9077, respectively).

### 3.4. CKD-Induced LV Hypertrophy in Male and Female Mice

To assess cardiac hypertrophy, heart and LV weights were compared between groups and sexes. A sex-dependent difference was observed in the Control group, with male mice having larger heart weights than females at each time point (Supplementary Table S1). Heart weight increased at weeks 9 and 12 when compared to previous time points in both male and female mice from the Adenine group (Supplementary Table S1). Adenine males exhibited significantly lower LV weights at weeks 3, 6, and 9 when compared to the Control group, but no difference between these groups was observed at week 12 (Figure 3C). Adenine females exhibited significantly lower LV weight when compared to Control females at week 9 only (Figure 3C). Similar to heart weight, a sex-dependent difference in LV weight was observed between male and female Controls for weeks 6, 9, and 12 (Figure 3C). LV weight in Adenine mice was significantly higher at week 12 compared to other time points, increasing from 64.11 mg to 79.05 mg (123.3%) in males and from 61.37 mg to 72.56 mg (110.2%) in females between weeks 9 and 12 (Figure 3C). A sex-dependent difference in LV weight in Adenine mice was detected at week 6 only (Figure 3C). No statistical differences in LV weight were observed based on regimen duration in the Control group (Supplementary Figure S1B).

Echocardiography was also used to assess LV remodeling and hypertrophy (Table 1 and Figure 3D,E). A trending significant difference (*p* = 0.074) in systolic left ventricular posterior wall (LVPW;s) thickness was observed at week 12 in Adenine-fed males when compared to Control males (Figure 3D). Male sex and the adenine regimen were identified as factors contributing to the increase in LVPW;s size (Figure 3D). Adenine males demonstrated an increase in LVPW;s thickness at week 12 when compared to earlier time points, indicative of LV remodeling (Supplementary Figure S1C). A sex-dependent difference in the Adenine group was identified at week 12, with males showing a thicker LVPW;s when compared to Adenine females (Figure 3D).

To account for the smaller heart size in the Adenine group, the LV wall thickness (LVAW + LVPW) was normalized to total LV size (LVAW + LVPW + LVID), defined as the sum of LV anterior wall (LVAW), LVPW, and LV inner diameter (LVID). Adenine males exhibited a sex-dependent difference in LV thickness ratio (systole) at weeks 9 and 12 (Figure 3E). At week 12, LV thickness in Adenine males was 120.18% greater than in Control males and 135.64% greater than in Adenine females. Additionally, the LV thickness ratio in Adenine males significantly increased at weeks 9 and 12 when compared to previous time points of 3 and 6 weeks (Supplementary Figure S1D).

### 3.5. Male Sex Required for Manifestation of CKD-Induced LV Systolic and Diastolic Dysfunction

**Figure 4:**
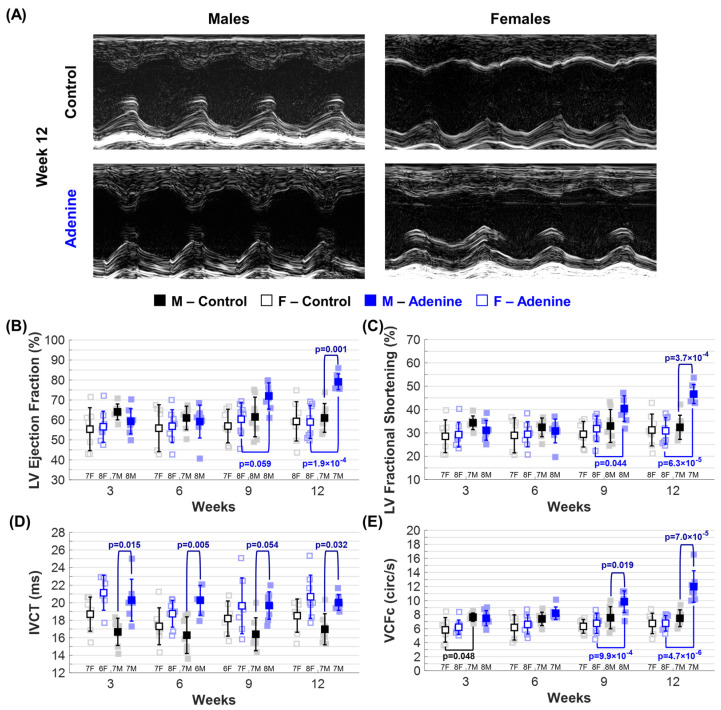
*Echocardiographic evaluation of left ventricular (LV) systolic function.* Changes in ■ male and □ female mice throughout progression of either **Control** (**Healthy**) or **Adenine** (**CKD**) diet regimen: (**A**) Representative M-mode images of LV chamber acquired in parasternal short-axis view. Echocardiogram-based parameters of systolic function tracked throughout disease progression included (**B**) LV ejection fraction, (**C**) LV fractional shortening, (**D**) isovolumetric contraction time (IVCT), and (**E**) velocity of circumferential fiber shortening corrected for heart rate (VCFc). Results are presented as mean ± standard deviation. The statistical significance of differences at each timepoint, considering sex (■ male and □ female) and regimen type (Healthy–Control or CKD–Adenine), was assessed using two-way ANOVA (Bonferroni) and shown with a navy-blue *p*-value. If a sex-dependent interaction is present at any time point, it is shown with black *p*-values for the Control group and blue *p*-values for the Adenine group.

Echocardiography was used to assess LV systolic, diastolic, and overall cardiac function (Table 1). Only Adenine males exhibited signs of LV systolic dysfunction throughout disease progression, with significant differences due to regimen type and sex. Adenine males had higher LV ejection fraction (Figure 4B) and fractional shortening (Figure 4C) at week 12 compared to both age-matched Control males and Adenine females. Additionally, both parameters increased throughout disease progression and were significantly higher in Adenine males at weeks 9 and 12 compared to earlier time points (Supplementary Figure S2A). Isovolumetric contraction time (IVCT), which reflects the period of LV contraction, was increased in Adenine males at all time points (Figure 4D), although not reaching statistical significance at week 9 (*p* = 0.054). However, IVCT did not change throughout disease progression (Supplementary Figure S2C). Velocity of circumferential fiber shortening (VCF) is another method used to estimate LV contractile function by measuring myocardial performance [26], and can be derived by the standard equation VCF = (LV − FS)/(LV − ET) (FS, LV Fractional Shortening; ET, LV Ejection Time). VCF is often corrected for HR to reflect the heart’s contractile function independently of rate variations. To do this, ET is corrected for HR (ETc) by dividing ET by the square root of the R-R interval (ET_c_ = ET/√R − R), with the R-R interval derived from echocardiogram-measured HR and expressed in ms. The HR-corrected velocity of circumferential fiber shortening (VCF_c_) is then calculated as VCF_c_ = (LV − FS)/(LV − ET_c_). Similarly to EF and FS, adenine males showed a regimen- and sex-dependent difference in VCF_c_ at weeks 9 and 12 compared to both Control males and Adenine females (Figure 4E).

**Figure 5:**
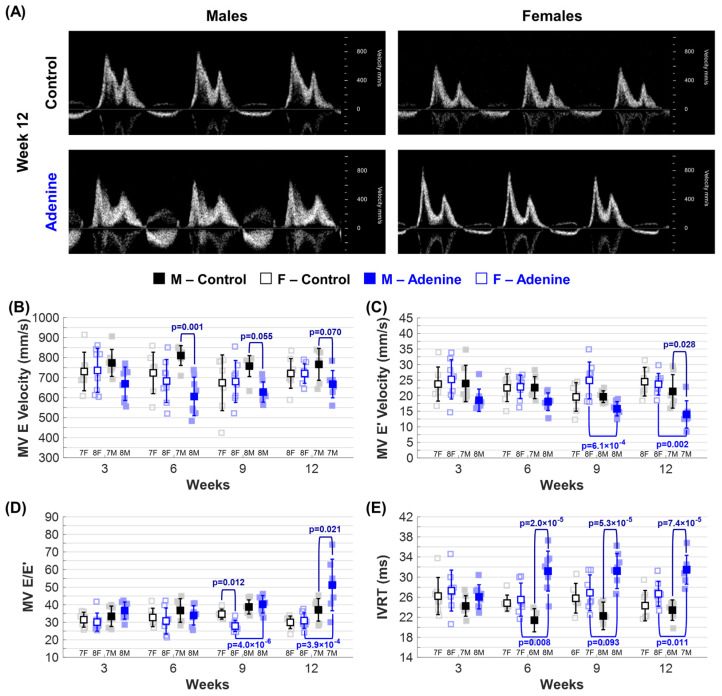
*Echocardiographic evaluation of left ventricular (LV) diastolic function.* Changes in ■ male and □ female mice throughout progression of either **Control** (**Healthy**) or **Adenine** (**CKD**) diet regimen: (**A**) Representative Pulse-Wave Doppler mitral valve (MV) flow images acquired in apical four-chamber view. Echocardiogram-based parameters of LV diastolic function tracked throughout disease progression included (**B**) mitral valve early flow velocity (MV E), (**C**) mitral annulus velocity (MV E′), (**D**) MV E/E′, and (**E**) isovolumetric relaxation time (IVRT). Results are presented as mean ± standard deviation. The statistical significance of differences at each timepoint, considering sex (■ male and □ female) and regimen type (Healthy–Control or CKD–Adenine), was assessed using two-way ANOVA (Bonferroni) and shown with a navy-blue *p*-value. If a sex-dependent interaction is present at any time point, it is shown with black *p*-values for the Control group and blue *p*-values for the Adenine group.

Mitral valve early flow velocity (MV E) decreased in Adenine males, with a significant difference observed at week 6 and a trend toward significance at weeks 9 and 12 (Figure 5B). At week 12, diastolic dysfunction associated septal mitral annulus velocity (MV E′) was significantly decreased in Adenine males compared to age-matched Control males and Adenine females (Figure 5C). Sex-dependent differences in MV E′ can also be observed between male and female mice in the Adenine group at week 9 (Figure 5C). Regimen- and sex-dependent differences were also observed at weeks 9 and 12 in the ratio of E to E′, defined as the MV E/E′ ratio (Figure 5D). The MV E/E′ ratio gradually increased through disease progression in Adenine males only (Supplementary Figure S3C). A significant increase in isovolumetric relaxation time (IVRT) was observed in Adenine males as early as week 6, which was significantly different from both Control males and Adenine females (Figure 5E). These changes coincide with CKD development, corresponding to the time required to observe CKD dysfunction in this mouse model [10]. An increase in MV E/E′ ratio and IVRT, as well as a decrease in MV E′ velocity, are all indicators of diastolic dysfunction in Adenine males. There was no significant difference in parameters of diastolic dysfunction in female mice due to disease manifestation nor progression at any time point (Supplementary Figure S3A–D).

### 3.6. Cardiac Performance Exacerbated by Male Sex in CKD Mouse Model

**Figure 6:**
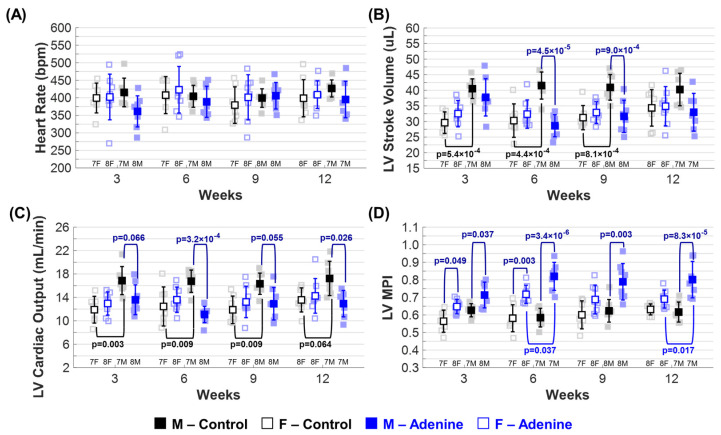
*Echocardiographic evaluation of overall left ventricular (LV) function.* Changes in ■ male and □ female mice throughout progression of either **Control** (**Healthy**) or **Adenine** (**CKD**) diet regimen. Echocardiogram-based parameters of overall LV function tracked throughout disease progression included (**A**) heart rate (bpm, beats per minute), (**B**) stroke volume, (**C**) LV cardiac output, and (**D**) LV myocardial performance index (MPI). Results are presented as mean ± standard deviation. The statistical significance of differences at each timepoint, considering sex (■ male and □ female) and regimen type (Healthy–Control or CKD–Adenine), was assessed using two-way ANOVA (Bonferroni) and shown with a navy-blue *p*-value. If a sex-dependent interaction is present at any time point, it is shown with black *p*-values for the Control group and blue *p*-values for the Adenine group.

Parameters indicative of overall LV function can be found in Table 1. Adenine males had significantly lower systolic (s) and diastolic (d) LV volumes at weeks 9 and 12 when compared to Control males, with sex-dependent significant differences from Adenine females observed at week 12 (Table 1). There were no significant differences in echocardiogram-measured HR due to regimen type or sex (Figure 6A). Control females had a lower stroke volume than male Controls at weeks 3, 6, and 9 (Figure 6B). Adenine males also exhibited a lower stroke volume when compared to Control males at weeks 6 and 9 (Figure 6B). Adenine females did not show changes in stroke volume either due to regimen type (Figure 6B) or over time (Supplementary Figure S4B). Control males showed an increase in cardiac output throughout the regimen duration at each time point compared to Control females (Figure 6C). Adenine males exhibited a decrease in cardiac output at each time point when compared to Control males but did not differ from Adenine females (Figure 6C). This decrease in cardiac output in Adenine males was consistent throughout the regimen duration, remaining stable despite disease progression (Supplementary Figure S4C). Male and female mice in the Adenine group showed changes in LV myocardial performance index (MPI), an index that correlates negatively with global LV function [27]. Compared to their same-sex Control counterparts, Adenine males showed a statistically significant increase in LV MPI at all time points, while Adenine females showed this increase only at weeks 3 and 6 (Figure 6D). There were no statistical changes in LV MPI in either sex due to disease progression (Supplementary Figure S4D).

### 3.7. CKD Increases Cycle Duration and Decreases HR in ECG Measurements

**Figure 7:**
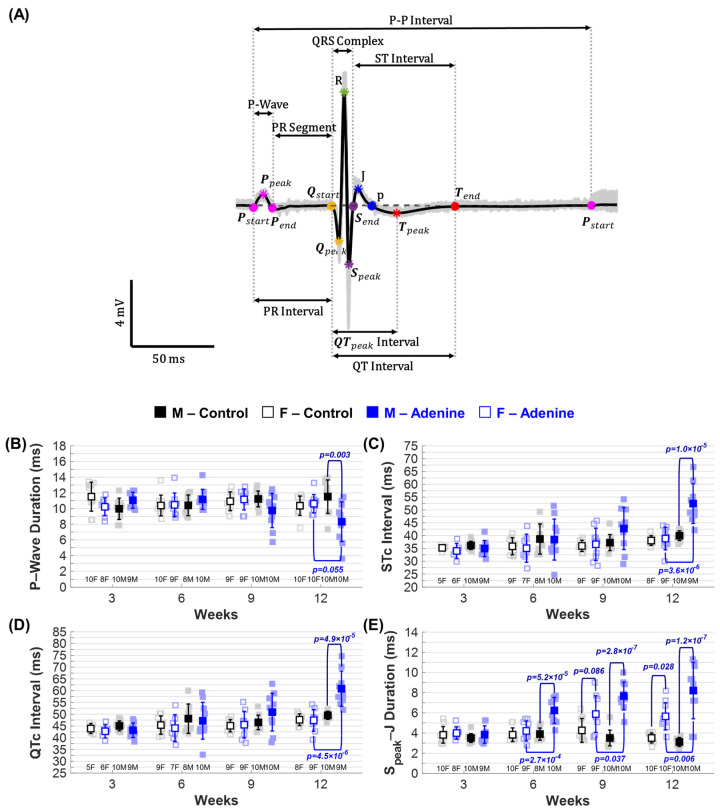
*Electrocardiogram (ECG) signal characteristics and changes in ECG parameters due to regimen type and duration.* Cardiac electrophysiological changes in ■ male and □ female mice measured via ECG and quantified using Lead I throughout progression of either **Control** (**Healthy**) or **Adenine** (**CKD**) regimen: (**A**) Mouse ECG waveform (Lead I) with its corresponding characteristics and intervals identified to quantify ECG parameters. ECG-based parameters tracked throughout disease progression included (**B**) P-wave duration, (**C**) QTc interval duration, (**D**) STc interval duration, and (**E**) S_peak_–J duration. Results are presented as mean ± standard deviation. The statistical significance of differences at each timepoint, considering sex (■ male and □ female) and regimen type (Healthy–Control or CKD–Adenine), was assessed using two-way ANOVA (Bonferroni) and shown with a navy-blue *p*-value. If a sex-dependent interaction is present at any time point, it is shown with black *p*-values for the Control group and blue *p*-values for the Adenine group.

Figure 7A illustrates the key features identified in an ECG signal used to quantify the duration of ECG parameters. Representative ECG tracings are shown in Supplementary Figure S5A, and the average duration of ECG parameters per regimen type and sex at each time point are listed in Table 2. Both RR interval and PP interval durations, which measure the average duration of one cardiac cycle, are significantly increased in the Adenine groups (Table 2). Compared to their Control counterparts, Adenine females showed an increase in cycle duration at weeks 9 and 12, while Adenine males showed an increase at all time points (Table 2). A sex-dependent difference between Adenine male and female mice was only identified at week 6 of the regimen (Table 2). No significant difference in cycle duration due to sex or regimen duration was identified for Control mice. HR was estimated using RR and PP interval durations. Since HR is inversely correlated with cycle duration, the Adenine regimen had the same influence on HR as it did on cycle duration. Significant differences in HR estimation between groups, per sex type were present, with Adenine mice showing an increase HR at all time points compared to Controls and a sex-dependent group difference (Table 2). No significant difference in HR was observed due to sex or regimen duration for Control mice.

### 3.8. Accounting for HR Variability in the Duration of ECG Parameters

Supplementary Table S3 shows the correlation coefficient of all identified ECG characteristics to RR interval duration based on regimen type. The PP interval duration had a strong correlation with RR interval duration (R = 0.9813), which is expected since both parameters measure the cycle duration from the start of one cycle to the next. Outside of PP interval duration, the QT interval duration had the highest correlation coefficient to RR interval duration for both the Control (R = 0.7293) and Adenine groups (R = 0.6552), with both groups having a combined R correlation coefficient of 0.7729 (Supplementary Table S3). The following parameters had an R value greater than 0.70 and were corrected (c) for cycle duration: Q–T_peak_, QT interval, R–T_peak_, S_peak_–T_peak_, ST interval, and J–T_peak_ (Supplementary Table S3). To ensure that HR variability was corrected for these parameters after using the correction formula, the linear correlation of the corrected parameters to RR interval duration was identified (Supplementary Table S4).

### 3.9. Increases in QTc and STc Interval Durations Depend on Sex, Regimen, and HR Correction

Differences in the duration of common ECG parameters were observed due to sex and regimen type. At week 12, only the Adenine males had a significantly shorter P-wave duration compared to the Control males, with no sex-dependent differences when compared to the Adenine females (Figure 7B). There were no significant differences in PR interval duration due to regimen type at any time point, and a sex-dependent difference was only observed between Adenine males and females at week 9 (Table 2). The QRS complex also showed no significant differences due to sex or regimen type at any time point. Both QTc and STc interval durations show a trend of increasing throughout disease progression in Adenine males (Supplementary Figure S5C,D), but significant differences are only observed at week 12 in comparison to Control males and Adenine females (Figure 7C,D). Without HR correction, an increase in QT and ST interval duration is also observed in Adenine females at weeks 9 and 12, with a sex-dependent difference when compared to Adenine males at week 12 only (Table 2). At week 12, adenine males showed a much larger difference compared to their sex-matched Controls and Adenine females—QT duration increased by 46.37% in males and 23.82% in females, while ST duration increased by 61.95% in males and 38.14% in females.

### 3.10. Sex-Specific Differences in S_peak_–J Duration Throughout CKD Disease Progression

To determine whether new ECG parameters can identify sex- and disease-dependent differences in cardiac electrophysiology, we measured the duration of various combinations of ECG markers. Of all the parameters identified, S_peak_–J duration was the only one that showed a trend throughout disease progression, with significant differences between regimen type and sex (Figure 7E). The duration of S_peak_–J differs based on sex and disease manifestation, with Adenine males exhibiting a statistically significant increase at week 6 and Adenine females at week 12 compared to Controls (Figure 7E). Adenine males were significantly different from Control males at weeks 6, 9, and 12, while Adenine females were only significantly different from their control counterparts at week 12 (Figure 7E). A trending significant difference was observed between Adenine males and females at week 9 (*p* = 0.086) (Figure 7E). Adenine males, on average, showed an increase in S_peak_–J duration throughout disease progression, but significant differences were only identified at week 12 when compared to previous time points (Supplementary Figure S5E). No significant differences in S_peak_–J duration were found due to sex or regimen duration for Control mice (Supplementary Figure S5E).

## 4. Discussion

Male and female sex affect physiological responses to CKD differently, influencing the progression and severity of both CKD and CKD-associated CVD [3]. Understanding how these differences influence cardiac structural, functional, and electrophysiological abnormalities can lead to more personalized approaches to managing and preventing cardiovascular complications in CKD patients. Additionally, recognizing sex-specific variations could enhance the accuracy of diagnostic tools and improve the prognostic value of existing and emerging biomarkers. This study was conducted to elucidate changes in CKD-induced cardiac dysfunction, with a particular emphasis on identifying sex-dependent variations in diagnostic CVD markers.

While the presence of cardiac hypertrophy in Adenine-treated males has been well reported [12,28,29,30], females are largely excluded from these studies, leaving it unclear whether this model also induces cardiac remodeling in female mice and whether sex-dependent differences exist. Chen et al. showed that male mice on a 16-week adenine-induced CKD regimen had increased cardiac fibrotic area and increases in both LVAW and LVPW thickness, which was also observed in our mice (Figure 3D) [29]. They also observed significantly lower body and heart weights in Adenine males, similar to our findings (Figure 3B and Supplementary Table S2) [12]. However, females were excluded from their study. Our results suggest that CKD-related cardiac structural remodeling occurs in both male and female mice in this Adenine-treated model, with notable sex-specific changes. We observed a larger increase in LV weight from week 9 to 12 in adenine-treated males (123.3%) compared to females (110.2%), suggesting that LV remodeling may progress more rapidly in males than in females (Figure 3C). Echocardiogram results showed that LV hypertrophy is a sex-dependent maker of CVD in Adenine males, which had an increase in LVPW;s thickness and LV wall thickness when compared to Control males and Adenine females (Figure 3D,E). These changes in LV wall thickness differ based on regimen type and sex, with Adenine males exhibiting features of hypertrophic cardiomyopathy. Future studies should assess whether adenine-treated females also exhibit cardiac fibrosis, as reported in adenine-treated males [29], and determine if remodeling response varies by sex.

A sex-dependent difference was also identified in echocardiogram-based measurements of cardiac function. Adenine males showed a decrease in both systolic and diastolic LV volume at week 12 compared to Control males and Adenine females (Table 1). Adenine females, despite having a smaller LV as measured by their weight (Figure 3C), maintained both LV volume (Table 1) and stroke volume (Figure 6B) throughout regimen duration. These morphological changes in LV volume in Adenine males account for the observed increases in LV ejection fraction and fractional shortening at week 12 (Figure 4B). Ejection fraction, calculated as the percentage of blood ejected from the ventricle during systole relative to the total amount of blood present in the ventricle at the end of diastole, can be affected by changes in LV wall thickness and chamber size. Patients with hypertrophic cardiomyopathies often exhibit an increase in ejection fraction due to maladaptive cardiac remodeling and reduced LV volume [31]. The same holds true for fractional shortening—thicker LV walls reduce the LV cavity size, causing exaggerated contractions and high fractional shortening, even if overall cardiac function is not compromised. We also observed a decrease in cardiac output in Adenine-treated males compared to Control males at all time points, with near significance (*p* < 0.07) across all weeks (Figure 6C). A reduced cardiac output with increased ejection fraction can also indicate HF with preserved ejection fraction, a condition where the heart muscle is stiff and struggles to relax during filling, leading to normal or even high ejection fraction despite reduced cardiac output due to impaired filling. Female sex is a risk factor for HF with preserved ejection fraction, particularly in the CKD population [32]. However, only Adenine males showed markers indicative of HF with preserved ejection fraction. This may be because adenine-treated males develop CKD earlier and with greater severity than females (Figure 2). More work would be needed to determine if adenine-treated mice can be used to model HF with preserved ejection fraction, including the characterization of common pathophysiological features. For murine models of HF with preserved ejection fraction, this should include tests to assess diastolic dysfunction, exercise intolerance, pulmonary edema, and concentric cardiac hypertrophy [33,34].

Increases in cardiac contractility have also been shown to increase ejection fraction [35]. At all regimen time points, Adenine males exhibited a prolonged IVCT when compared to Controls, but no sex-dependent differences were identified when compared to Adenine females (Figure 4D). Although Adenine females also had an average increase in IVCT at weeks 3, 9, and 12, IVCT was not statistically significant from Control females (Figure 4D). A prolonged IVCT strongly correlates with LV dyssynchrony, a condition that affects electromechanical activation of cardiac muscles during systole and leads to a decrease in cardiac output [36]. Increases in VCFc also suggest a sex-dependent preference for contractile abnormalities in adenine-treated males (Figure 4E). Similarly to increased LV ejection fraction and fractional shortening, higher VCFc reflects more forceful or efficient ventricular contraction, indicating improved systolic function. However, elevated VCFc can also occur in pathological conditions like hypertrophic cardiomyopathy, where thicker LV walls enhance contractility due to structural changes in the myocardium [37]. Changes in VCFc exhibit a similar trend to those of LV ejection fraction and fractional shortening and have been well reported in several mouse models of cardiac disease [38,39,40]. However, no previous studies have reported changes in VCFc related to sex and the adenine regimen. The identified increases in LV ejection fraction, fractional shortening, IVCT, and VCFc indicate the presence of systolic dysfunction in the adenine-treated mouse model, with significant differences observed only in male mice. Echocardiogram-measured parameters associated with diastolic dysfunction (i.e., HF with preserved ejection fraction) also demonstrated dependence on male sex and regimen duration. As early as week 6, Adenine males showed a significant disease- and sex-dependent increase in MV E/E′ and IVRT (Figure 5D,E), both of which are common markers used to identify diastolic dysfunction in mice [33,41]. These data suggest that male sex is essential for the occurrence of systolic and diastolic dysfunction in this adenine-treated mouse model.

The QTc and STc intervals were notably prolonged in adenine males at week 12, correlating with increased cardiac dysfunction. The QTc interval is a measure of cardiac depolarization and repolarization that highly correlates to ventricular arrythmias and sudden cardiac death. In CKD patients, an elongated QTc is associated with higher risks of CVD events, as well as all-cause and CVD mortality [42,43,44]. A study by King et al. also found a prolonged QT interval in mice fed a high (25%) and low (15%) dose dietary adenine regimen with an alternating control diet for 6 weeks [30]. More importantly, they found that changes in QT duration in CKD mice are associated with severity of CKD and are sex-dependent, with Adenine males showing worse renal dysfunction and cardiac conduction than Adenine females [30]. This study, however, did not assess cardiac functional changes nor correct the QT interval for heart rate throughout disease progression. In our study, correction for HR was necessary to accurately assess sex-dependent differences in the QT interval. It has been well documented that a sex difference exists in QTc interval durations between men and women, with women showing a longer QTc than men [45,46,47]. Further complicating matters, changes in endogenous sex hormones throughout the menstrual cycle have been shown to influence QT interval in both humans and animals [48,49,50]. Age- and sex-specific cutoffs for prolonged QTc have been proposed to improve its prognostic value [51]. These sex-specific differences in the QT interval emphasize the importance of considering HR adjustments in ECG analysis, particularly in mouse models that significantly affect HR.

A new ECG parameter, S_peak_–J duration, showed significant sex- and disease-dependent differences, especially in Adenine males. A prolonged and statistically different S_peak_–J duration was observed starting at week 6 of the regimen in Adenine males, but only at week 12 in Adenine females (Figure 7E). In mice, the myocardium activates early (R-wave) or late (J-wave) with a large repolarization phase, which then plateaus until the p marker and then continuously decreases until the start of the P-wave [17]. Therefore, a prolonged S_peak_–J duration is indicative of abnormal early repolarization periods. In humans, alterations in J-wave morphology are also indicative of early repolarization issues and have been linked to a higher risk of ventricular arrhythmias and sudden cardiac death [52]. Unlike in mice, a J-wave is slightly visible in humans and depends on lead placement, with a prominent J-wave appearing mostly in pathophysiological conditions [20,53,54]. Due to this, the quantification of ECG parameter durations involving this wave has not been well studied in humans. The prevalence of J-waves indicative of early repolarization has been shown to increase in CKD patients, but unlike with the general population, did not seem to correlate to all-cause mortality [55]. Further studies are needed to correlate the electrophysiology of the J-wave between species and assess the potential of using J-wave-dependent ECG parameters to detect cardiac electrophysiological changes in CKD patients.

While cardiac morphological and functional changes have been widely studied in the 5/6 nephrectomy mouse model of CKD [18,56], only a few studies have reported these changes in the adenine-induced CKD mouse model. Cardiac structural and functional changes in this adenine-treated mouse model were first reported in 2018, making it a relatively new mouse model to study reno-cardiac syndrome [12]. Although more studies exist on the 5/6 nephrectomy mouse model of CKD-induced CVD, very few include female mice. A recent systematic review by Soppert et al. revealed that 71% of studies on uremic cardiomyopathy used male mice, with only 6% analyzing both sexes [57]. Despite the known importance of sex in the development of CKD and CKD-induced cardiovascular disease, there is a lack of studies that account for sex differences. In our study, the functional changes observed suggest that males experience significantly worse cardiac dysfunction compared to females. This may be due to the fact that males exhibit more severe CKD, as indicated by increased BUN levels (Figure 2) and previously reported by another group [30], thus exacerbating cardiac issues. Since CKD progresses faster in males than in females, the onset of CKD-induced CVD may also occur earlier in males. Therefore, sex-specific differences in CKD severity are most likely a major contributing factor to the observed disparity in cardiac function in this mouse model. Since a similar sex disparity in CKD progression is observed in humans, we believe this animal model is still suitable for studying sex differences in CKD-induced cardiac dysfunction. Considering that we observed sex-dependent changes in LV hypertrophy, a common morphology that can predict the risk of adverse cardiovascular events in CKD patients, and that sex differences exist in the manifestation and progression of CKD in this mode and in humans, with female sex being a protective factor in CKD-associated CVD risk, we conclude that an advantage of this model is its ability to study these CKD-induced CVD sex differences [58,59]. However, to ensure that the observed differences are due to sex and not CKD progression, it may be more beneficial to compare the sexes when CKD severity is similar, rather than based on regimen duration. CKD severity can be assessed by measuring blood chemistries, such as urea nitrogen and creatinine levels, as well as by quantifying kidney fibrosis. Other important factors to consider when studying sex differences are sex chromosomes and hormone influence, which were not considered in this study. The four core genotypes model [60], which considers the sex chromosome complement independent of the animal’s gonadal sex, could be used to further explore sex-dependent factors in this adenine-fed model.

Although not tracked in this study, one of the disadvantages of this mouse model is the high mortality rate at later stages of disease. We observed that over 50% of the Adenine males died before reaching week 12 of the adenine regimen, while females survived over 95% of the time. Mortality rates of adenine-induced CKD mouse models in the literature are contradictory. The study by Chen et al., which excluded females, found that 100% of Adenine-fed (0.20%) males survived after a 16-week regimen [29]. Kim et al. found that 100% of the male and female mice survived when alternating a high adenine (0.15–0.20%) and casein diet within a 10-week period [61]. Padalkar et al. adjusted the amount of adenine in the diet based on sex to try to reduce mortality in male mice, with males receiving an adenine dose of 0.1% and females 0.2% [62]. However, they still showed that males on a 0.1% adenine diet had a much lower survival rate than females on a 0.2% adenine diet, with ~20 to 25% of males surviving compared to ~90% of females [62]. The reported differences in mortality rates are most likely influenced by experimental design, with the type of adenine used to supplement normal diet, adenine dosage, alternating between an adenine-enriched and normal diet, and regimen duration all contributing to these discrepancies.

Another important contributing factor to consider that could contribute to sex-dependent differences in mortality is the progression of both CKD and CKD-induced CVD. Our results show that male sex plays an important role in the manifestation and progression of CKD and CKD-induced CVD, with Adenine males exhibiting more advanced renal failure and significant changes in cardiac structural, functional, and electrophysiological markers of disease. Adenine males had a shorter P-wave duration, which has been associated with increased risks of atrial fibrillation (Figure 6B,C) [63]. Patients with CKD and atrial fibrillation have a significantly increased risk of myocardial infarction and other adverse cardiac events, with females being at higher risk compared to men [64,65]. As previously discussed, the adenine-induced CKD mouse model shows a prolonged QTc duration with sex-dependent differences in the progression of CKD, with female sex being protective against CKD [30]. The same study also showed that CKD mice had an increased risk of sudden cardiac death due to progressive bradyarrhythmias but did not specify whether this risk varied by sex [30]. This phenomenon is also observed in humans, with males progressing faster and having a higher incidence of end-stage kidney disease than females despite females having a higher incidence of CKD [66,67]. We hypothesize that this sex-dependent difference in CKD and CKD-induced CVD progression also contributes to mortality rate, with Adenine males progressing in both diseases faster than females. As a result, adenine-treated males may be at greater risk of experiencing cardiovascular-related events (e.g., myocardial infarction and sudden cardiac death) and, consequently, death.

In addition to an increased mortality rate, Adenine males significantly lose body weight, with Adenine males being less than 50% of the size of sex-matched Controls (Figure 3B,C). This drastic reduction in body weight can falsely exaggerate changes in other cardiac structures if used for their normalization. To prevent this but still account for changes in body size, we tried normalizing cardiac structure to heart weight. Normalization of LV weight to heart weight only showed significant differences between Adenine and Control males at week 3 (Supplementary Table S2). Control males had a lower LV/Heart weight ratio at week 3 compared to other weeks (Supplementary Table S2). Reduction in body size, as well as the progression of disease, greatly affect procedures requiring anesthesia. This also interferes with the acquisition of echocardiograms, as the ribs become more pronounced and the heart twists severely to the left as body weight is reduced, making it difficult to visualize and evaluate cardiac function. Further studies are needed to determine the best type of adenine regimen to elucidate sex-dependent differences in CKD-induced CVD progression while reducing the complications associated with this model.

## 5. Conclusions

In this study, we identified sex-dependent differences in the progression of CKD and CKD-induced CVD in an Adenine-treated mouse model. We observed significant changes in cardiac structure, function, and electrophysiology based on sex and regimen duration. We identified that Adenine-treated males develop CKD earlier and with greater severity than females. We determined that male sex is crucial for the development of LV hypertrophy, LV systolic dysfunction, and LV diastolic dysfunction. We showed a regimen-, duration-, and sex-dependence in the duration of common ECG parameters, with Adenine males exhibiting an increase in QTc duration compared to Control males and Adenine females at late stages of the disease, and that cardiac functional changes correlate to ECG characteristics. More importantly, we identified a new ECG parameter (Speak-J duration) that can identify sex-specific cardiac electrophysiological changes in Adenine mice. The findings in this study show the feasibility of using this mouse model of CKD-induced CVD to study sex-dependent differences in cardiac structure, function, and electrophysiology.

## Figures and Tables

**Table 1:** *Echocardiographic measures of cardiac function.* Cardiac structural, functional, and hemodynamic changes measured via echocardiography in male and female mice fed either a **Control** (**Healthy**) or **Adenine** (**CKD**) diet for up to 12 weeks.

	Week 3						Week 6						Week 9						Week 12					
	Male			Female			Male			Female			Male			Female			Male			Female		
Parameters	Control	Adenine		Control	Adenine		Control	Adenine		Control	Adenine		Control	Adenine		Control	Adenine		Control	Adenine		Control	Adenine	
	*n* = 7M	*n* = 8M	*p*-Value	*n* = 7F	*n* = 8F	*p*-Value	*n* = 6–7M	*n* = 6–8M	*p*-Value	*n* = 6–7F	*n* = 8F	*p*-Value	*n* = 8M	*n* = 8M	*p*-Value	*n* = 6–7F	*n* = 8F	*p*-Value	*n* = 6–7M	*n* = 7M	*p*-Value	*n* = 7–8F	*n* = 8F	*p*-Value
***LV Remodeling***																								
LVAW;s (mm)	1.28 ± 0.11	1.33 ± 0.14	1.0	1.08 ± 0.17	1.15 ± 0.16	1.0	1.25 ± 0.11	1.23 ± 0.16	1.0	1.11 ± 0.11	1.17 ± 0.15	1.0	1.26 ± 0.16	1.46 ± 0.19	0.113	0.97 ± 0.41	1.21 ± 0.14** §**	1.0	1.34 ± 0.18	1.40 ± 0.09	1.0	1.29 ± 0.10	1.18 ± 0.14** §**	0.475
LVAW;d (mm)	0.82 ± 0.10	0.92 ± 0.12	0.712	0.71 ± 0.11	0.76 ± 0.09	1.0	0.76 ± 0.11	0.85 ± 0.15	0.959	0.76 ± 0.12	0.83 ± 0.11	1.0	0.78 ± 0.09	0.88 ± 0.15	0.415	0.64 ± 0.27	0.80 ± 0.10	1.0	0.90 ± 0.11	0.82 ± 0.07	1.0	0.84 ± 0.10	0.77 ± 0.17	1.0
LVPW;s (mm)	1.14 ± 0.14	1.04 ± 0.10	0.812	0.97 ± 0.14	0.93 ± 0.14	1.0	1.03 ± 0.11	0.97 ± 0.17	1.0	0.93 ± 0.07	1.03 ± 0.11	0.756	1.02 ± 0.15	1.11 ± 0.18	1.0	0.84 ± 0.36	1.01 ± 0.10	1.0	1.14 ± 0.13	1.37 ± 0.25	0.074	1.03 ± 0.13	1.01 ± 0.11** §**	1.0
LVPW;d (mm)	0.72 ± 0.04	0.70 ± 0.06	1.0	0.68 ± 0.10	0.67 ± 0.06	1.0	0.73 ± 0.06	0.64 ± 0.10	0.497	0.69 ± 0.05	0.72 ± 0.13	1.0	0.70 ± 0.06	0.76 ± 0.14	1.0	0.60 ± 0.25	0.71 ± 0.13	1.0	0.77 ± 0.06	0.80 ± 0.06	1.0	0.73 ± 0.07	0.68 ± 0.12** §**	1.0
LVID;s (mm)	2.52 ± 0.17	2.66 ± 0.38	1.0	2.57 ± 0.35	2.62 ± 0.36	1.0	2.68 ± 0.22	2.40 ± 0.46	0.981	2.58 ± 0.40	2.60 ± 0.35	1.0	2.66 ± 0.46	1.96 ± 0.24	***0.003***	2.24 ± 0.96	2.48 ± 0.35** §**	1.0	2.65 ± 0.34	1.72 ± 0.18	***2.2 × 10^−5^***	2.55 ± 0.34	2.58 ± 0.30** §**	1.0
LVID;d (mm)	3.83 ± 0.13	3.85 ± 0.35	1.0	3.59 ± 0.15	3.69 ± 0.29	1.0	3.96 ± 0.16	3.45 ± 0.38	***0.009***	3.61 ± 0.23	3.68 ± 0.26	1.0	3.94 ± 0.30	3.29 ± 0.21	***1.1 × 10^−4^***	3.17 ± 1.30	3.62 ± 0.25	1.0	3.91 ± 0.26	3.21 ± 0.24	***8.2 × 10^−5^***	3.70 ± 0.23	3.73 ± 0.24** §**	1.0
LV Thickness;s	0.46 ± 0.06	0.47 ± 0.06	1.0	0.45 ± 0.07	0.44 ± 0.07	1.0	0.45 ± 0.04	0.47 ± 0.04	1.0	0.40 ± 0.06	0.43 ± 0.09	1.0	0.47 ± 0.06	0.57 ± 0.03	***0.003***	0.44 ± 0.06	0.48 ± 0.06** §**	1.0	0.48 ± 0.05	0.61 ± 0.04	***1.5 × 10^−4^***	0.48 ± 0.05	0.46 ± 0.05** §**	1.0
LV Thickness;d	0.29 ± 0.01	0.30 ± 0.03	1.0	0.28 ± 0.02	0.28 ± 0.03	1.0	0.27 ± 0.02	0.30 ± 0.04	0.349	0.29 ± 0.03	0.30 ± 0.03	1.0	0.27 ± 0.03	0.33 ± 0.03	***0.002***	0.28 ± 0.02	0.29 ± 0.04	1.0	0.30 ± 0.02	0.34 ± 0.02	0.191	0.30 ± 0.03	0.28 ± 0.04** §**	1.0
***LV Systolic Function***																								
EF (%)	64.0 ± 4.0	59.3 ± 6.3	1.0	55.4 ± 10.7	56.6 ± 7.8	1.0	61.0 ± 5.9	59.2 ± 8.2	1.0	55.9 ± 11.7	56.9 ± 8.3	1.0	61.5 ± 9.9	72.0 ± 6.6	0.112	49.8 ± 21.6	60.3 ± 8.3	1.0	61.0 ± 7.2	79.1 ± 3.9	***0.001***	59.3 ± 9.9	58.9 ± 8.3** §**	1.0
FS (%)	34.3 ± 2.9	31.1 ± 4.2	1.0	28.5 ± 7.0	29.3 ± 5.3	1.0	32.4 ± 4.0	30.8 ± 5.1	1.0	28.9 ± 7.5	29.4 ± 5.4	1.0	33.0 ± 7.0	40.4 ± 5.5	0.111	25.8 ± 11.6	31.8 ± 5.5** §**	1.0	32.4 ± 5.2	46.6 ± 4.1	***3.7 × 10^−4^***	31.2 ± 6.8	30.9 ± 5.7** §**	1.0
VCF_c_ (circ/s)	7.6 ± 0.6	7.5 ± 1.1	1.0	5.8 ± 1.8 **#**	6.2 ± 1.0	1.0	7.4 ± 1.0	7.2 ± 3.0	1.0	6.2 ± 1.8	6.6 ± 1.4	1.0	7.6 ± 1.6	9.8 ± 1.5	***0.019***	6.3 ± 1.0	6.8 ± 1.4** §**	1.0	7.5 ± 1.2	12.0 ± 2.2	***6.9 × 10^−5^***	5.9 ± 2.7	6.8 ± 1.1** §**	1.0
IVCT (ms)	16.7 ± 1.6	17.8 ± 7.5	***0.015***	18.7 ± 1.9	15.9 ± 9.9	0.229	16.3 ± 2.1	15.2 ± 9.5	***0.005***	17.3 ± 2.1	18.7 ± 1.5	0.906	14.4 ± 6.1	19.7 ± 1.6	0.054	13.7 ± 8.6	17.2 ± 7.5	1.0	17.0 ± 1.8	20.0 ± 0.9	***0.032***	16.2 ± 6.8	20.7 ± 2.5	0.209
***LV Diastolic Function***																								
MV E Velocity (mm/s)	773.6 ± 67.2	669. ± 83.5	0.215	730.6 ± 96.3	736.1 ± 109.3	1.0	810.4 ± 49.5	605.6 ± 96.1	***0.001***	723.7 ± 103.5	683.1 ± 107.5	1.0	757.6 ± 52.5	627.9 ± 50.8	0.055	590.0 ± 271.0	680.7 ± 104.9	1.0	766.2 ± 79.6	667.2 ± 68.1	0.070	720.9 ± 74.1	720.8 ± 48.7	1.0
MV E′ Velocity (mm/s)	23.9 ± 5.8	18.5 ± 3.6	0.382	23.7 ± 5.5	25.2 ± 6.3	1.0	22.6 ± 3.5	18.1 ± 2.8	0.151	22.5 ± 4.4	22.8 ± 3.8	1.0	19.7 ± 1.9	15.8 ± 2.3	0.384	17.2 ± 8.1	24.9 ± 5.9** §**	0.097	21.4 ± 5.4	14.0 ± 4.4	***0.028***	24.5 ± 4.6	23.7 ± 3.5** §**	1.0
MV E/E′	33.4 ± 5.8	36.7 ± 4.9	1.0	31.5 ± 4.3	30.1 ± 5.2	1.0	36.7 ± 6.7	33.9 ± 5.5	1.0	32.7 ± 5.3	30.7 ± 7.4	1.0	38.8 ± 4.0	40.3 ± 5.0	1.0	30.3 ± 12.5	27.9 ± 3.1** §**	***0.012***	37.1 ± 6.4	51.2 ± 14.6	***0.021***	29.9 ± 3.6	30.9 ± 4.5** §**	1.0
IVRT (ms)	24.3 ± 2.0	26.1 ± 2.4	1.0	26.2 ± 3.7	27.3 ± 4.1	1.0	18.3 ± 8.4	31.2 ± 4.0	***1.9 × 10^−5^***	24.8 ± 1.6	22.3 ± 9.5** §**	1.0	22.3 ± 2.7	31.2 ± 3.5	***5.2 × 10^−5^***	19.3 ± 12.2	23.5 ± 10.1	1.0	20.1 ± 9.0	31.5 ± 2.9	***7.4 × 10^−5^***	21.3 ± 9.0	26.7 ± 2.5** §**	0.579
***LV Overall Function***																								
Heart Rate (bpm)	415.4 ± 40.7	361.3 ± 44.3	0.273	399.5 ± 42.6	402.3 ± 65.1	1.0	404.4 ± 31.3	388.6 ± 44.3	1.0	407.6 ± 52.8	423.0 ± 66.4	1.0	399.2 ± 26.4	406.0 ± 37.9	1.0	331.8 ± 142.5	401.4 ± 64.4	1.0	426. ± 24.3	395.1 ± 51.9	1.0	398.9 ± 52.9	408.9 ± 39.4	1.0
Volume;s	22.9 ± 3.8	26.6 ± 8.1	1.0	24.7 ± 8.1	25.8 ± 8.5	1.0	26.8 ± 5.4	23.9 ± 12.8	1.0	24.9 ± 9.4	25.4 ± 8.3	1.0	27.1 ± 11.0	13.5 ± 4.8	***0.011***	21.3 ± 11.1	22.5 ± 8.2	1.0	28.3 ± 9.2	8.8 ± 2.2	***8.2 × 10^−5^***	24.1 ± 7.8	24.6 ± 6.7** §**	1.0
Volume;d	63.4 ± 5.2	69.5 ± 15.9	1.0	54.3 ± 5.5	58.4 ± 11.0	1.0	68.3 ± 6.5	56.2 ± 19.1	0.400	55.1 ± 8.4	57.8 ± 9.8	1.0	68.0 ± 12.2	46.3 ± 9.2	***5.9 × 10^−4^***	48.6 ± 21.1	55.4 ± 9.2	1.0	71.8 ± 17.8	41.7 ± 7.5	***1.6 × 10^−4^***	58.5 ± 8.4	59.5 ± 8.8** §**	1.0
Cardiac Output (mL/min)	16.9 ± 2.4	13.6 ± 2.5	0.066	11.9 ± 2.3 **#**	13.0 ± 2.0	1.0	16.8 ± 1.9	11.1 ± 1.4	***3.2 × 10^−4^***	12.5 ± 3.3 **#**	13.6 ± 2.2	1.0	16.3 ± 1.8	12.9 ± 2.8	0.055	10.4 ± 4.7 **#**	13.2 ± 2.7	1.0	17.2 ± 2.9	12.9 ± 2.2	***0.026***	13.6 ± 2.1	14.3 ± 3.0	1.0
Stroke Volume (µL)	40.5 ± 3.1	37.7 ± 6.0	1.0	29.6 ± 3.5 **#**	32.5 ± 4.2	1.0	41.5 ± 4.4	28.6 ± 3.5	***4.5 × 10^−5^***	30.3 ± 5.4 **#**	32.4 ± 4.5	1.0	40.9 ± 4.1	31.6 ± 5.2	***9.0 × 10^−4^***	27.3 ± 11.6 **#**	32.9 ± 3.5	1.0	40.2 ± 5.2	33.0 ± 6.1	0.173	34.4 ± 5.9	34.9 ± 6.3	1.0
ET	54.9 ± 3.1	54.5 ± 3.4	1.0	62.3 ± 9.8	57.3 ± 5.0	0.661	53.7 ± 4.0	42.2 ± 17.7	0.660	58.8 ± 8.8	53.5 ± 5.9	0.692	53.7 ± 4.2	50.3 ± 4.8	1.0	52.2 ± 21.3	60.1 ± 8.5** §**	1.0	51.1 ± 2.9	48.3 ± 6.3	1.0	57.0 ± 4.1	54.8 ± 3.4** §**	1.0
MPI	0.6 ± 0.0	0.7 ± 0.1	***0.037***	0.6 ± 0.1	0.6 ± 0.0	***0.049***	0.6 ± 0.1	0.7 ± 0.3	***3.3 × 10^−6^***	0.6 ± 0.1	0.7 ± 0.1** §**	***0.003***	0.6 ± 0.1	0.8 ± 0.1	***0.003***	0.5 ± 0.2	0.7 ± 0.1	0.324	0.6 ± 0.1	0.8 ± 0.1	***8.2 × 10^−5^***	0.6 ± 0.0	0.7 ± 0.1** §**	0.481

The background color of the columns is grey for the Control groups and blue for the Adenine group. Results are presented as mean ± standard deviation. Two-way ANOVA plus Bonferroni’s multiple comparisons correction was used to detect significance between groups at each time point, considering regimen type and sex, and shown with a navy-blue *p*-value. If a sex-dependent interaction exists (*p* < 0.05) at each time point, it is depicted as # (black) to the Control group and § (blue) in the Adenine group. Abbreviations used: LV, left ventricle;s, systole;d, diastole; LVAW, left ventricular anterior wall; LVPW, left ventricular anterior wall; LVID, left ventricular inner diameter; EF, ejection fraction; FS, fractional shortening; VCFc, velocity of circumferential fiber shortening, normalized to heart rate; IVCT, isovolumetric contraction time; MV E, mitral valve early flow velocity; MV E′, mitral annulus velocity; IVRT, isovolumetric relaxation time; bpm, beats per minute; ET, ejection time; MPI, myocardial performance index. LV thickness is defined as the ratio of LV wall size (LVAW + LVPW) to LV chamber size (LVAW + LVPW + LVID). Heart rate in this table was derived from LV trace measurements acquired in parasternal short-axis view using M-mode.

**Table 2:** *Duration of electrocardiogram (ECG) parameters.* Regimen- and sex-dependent differences in duration of ECG characteristics in male and female mice fed either a **Control** (**Healthy**) or **Adenine** (**CKD**) diet for up to 12 weeks.

	Week 3						Week 6						Week 9						Week 12					
	Male			Female			Male			Female			Male			Female			Male			Female		
Duration	Control	Adenine		Control	Adenine		Control	Adenine		Control	Adenine		Control	Adenine		Control	Adenine		Control	Adenine		Control	Adenine	
	*n* = 7–10M	*n* = 9M	*p*-Value	*n* = 5–10F	*n* = 6–8F	*p*-Value	*n* = 7–8M	*n* = 10M	*p*-Value	*n* = 9–10F	*n* = 7–9F		*n* = 9–10M	*n* = 10M	*p*-Value	*n* = 7–9F	*n* = 9F	*p*-Value	*n* = 10M	*n* = 9–10M	*p*-Value	*n* = 8–10F	*n* = 9–10F	*p*-Value
RR Interval (ms)	123.9 ± 9.3	149.4 ± 25.6	***0.015***	133.4 ± 9.2	137.3 ± 19.9	1.0	141.0 ± 14.7	202.5 ± 22.6	**8.0 × 10^−7^**	135.8 ± 20.9	140.5 ± 17.3** §**	1.0	133.4 ± 8.8	201.0 ± 21.2	***6.2 × 10^−7^***	134.6 ± 14.3	180.4 ± 36.8	***7.8 × 10^−4^***	120.8 ± 18.2	162.5 ± 41.7	***0.010***	116.2 ± 11.1	164.5 ± 28.1	***0.002***
HR–RR Interval (bpm)	486.9 ± 38.9	412.2 ± 70.5	**0.020**	451.8 ± 30.4	444.5 ± 60.0	1.0	429.7 ± 45.7	299.4 ± 31.4	***2.5 × 10^−5^***	450.7 ± 64.9	432.6 ± 50.8** §**	1.0	451.4 ± 28.8	301.6 ± 32.7	***9.7 × 10^−8^***	449.9 ± 43.0	345.1 ± 69.6	***1.5 × 10^−4^***	506.5 ± 73.4	391.7 ± 102.8	***0.008***	520.6 ± 48.1	374.0 ± 61.5	***5.3 × 10^−4^***
PP Interval (ms)	121.9 ± 8.1	143.3 ± 23.3	***0.033***	131.3 ± 8.8	133.6 ± 18.9	1.0	135.7 ± 16.1	197.7 ± 20.9	**3.2 *× 10*^−7^**	133.7 ± 20.3	136.1 ± 16.1** §**	1.0	130.4 ± 9.6	192.2 ± 21.7	***1.2 × 10^−6^***	132.3 ± 13.6	173.9 ± 33.4	***0.001***	119.4 ± 16.8	154.0 ± 30.5	***0.009***	114.0 ± 10.2	160.6 ± 27.0	***3.0 × 10^−4^***
HR–PP Interval (bpm)	494.3 ± 34.9	428.5 ± 68.9	***0.041***	459.0 ± 30.2	456.3 ± 58.7	1.0	447.7 ± 52.6	306.5 ± 30.5	***8.0 × 10^−6^***	457.4 ± 63.2	446.1 ± 50.7** §**	1.0	462.3 ± 32.1	315.8 ± 36.6	***2.1 × 10^−7^***	457.3 ± 43.1	356.3 ± 67.5	**3.1 *× 10^−4^***	511.5 ± 70.0	405.7 ± 93.2	**0.010**	529.9 ± 45.7	382.9 ± 61.6	***2.2 × 10^−4^***
P-Wave (ms)	10.0 ± 1.4	11.1 ± 1.0	0.607	11.5 ± 1.9	10.2 ± 1.1	0.359	10.4 ± 1.3	11.2 ± 1.3	1.0	10.4 ± 1.3	10.5 ± 1.5	1.0	11.2 ± 1.0	9.7 ± 2.2	0.223	10.9 ± 1.2	11.2 ± 1.3	1.0	11.5 ± 2.1	8.3 ± 2.5	***0.003***	10.4 ± 1.2	10.6 ± 1.2	1.0
PR Interval (ms)	37.3 ± 2.0	40.6 ± 3.1	0.098	40.4 ± 3.1	38.1 ± 3.1	0.607	40.5 ± 7.9	40.6 ± 4.5	1.0	38.9 ± 2.8	38.6 ± 1.2	1.0	40.6 ± 2.2	41.2 ± 4.1	1.0	39.7 ± 2.8	45.7 ± 5.2	***0.010***	39.4 ± 2.8	39.6 ± 9.5	1.0	38.9 ± 2.7	41.8 ± 4.2	1.0
PR Segment (ms)	27.3 ± 1.9	29.5 ± 2.7	0.335	28.9 ± 2.6	27.9 ± 2.6	1.0	30.1 ± 6.9	29.4 ± 3.9	1.0	28.5 ± 2.8	28.1 ± 1.9	1.0	29.4 ± 2.1	31.4 ± 2.7	1.0	28.8 ± 2.2	34.6 ± 5.5	***0.005***	27.9 ± 2.0	31.2 ± 8.0	0.763	28.6 ± 2.4	31.2 ± 4.1	1.0
Q_start_–R (ms)	6.3 ± 0.9	5.6 ± 1.3	0.937	6.2 ± 1.0	6.2 ± 0.5	1.0	6.6 ± 0.6	6.6 ± 0.8	1.0	6.8 ± 0.9	6.5 ± 0.7	1.0	6.8 ± 0.5	6.1 ± 1.3	1.0	6.3 ± 0.7	6.5 ± 1.7	1.0	6.5 ± 0.6	6.2 ± 0.7	1.0	6.4 ± 1.1	6.1 ± 1.3	1.0
Q_start_–S_peak_ (ms)	8.7 ± 1.0	8.3 ± 1.5	1.0	8.9 ± 1.3	8.7 ± 0.7	1.0	9.4 ± 0.8	9.1 ± 0.9	1.0	9.4 ± 1.1	9.0 ± 0.7	1.0	9.2 ± 0.7	8.4 ± 1.5	1.0	9.0 ± 0.9	9.1 ± 1.7	1.0	9.4 ± 0.8	8.7 ± 1.0	0.900	9.3 ± 1.3	8.6 ± 1.5	1.0
QRS Complex (ms)	11.2 ± 0.8	10.5 ± 1.8	1.0	11.1 ± 1.4	11.0 ± 0.5	1.0	11.9 ± 0.7	12.2 ± 1.7	1.0	11.4 ± 1.1	11.3 ± 1.2	1.0	11.5 ± 0.9	12.0 ± 3.0	1.0	12.4 ± 1.0	12.0 ± 2.2	1.0	11.2 ± 1.0	13.1 ± 2.7	0.211	10.9 ± 1.2	11.8 ± 2.4	1.0
QRSJ (ms)	12.3 ± 0.8	12.1 ± 1.7	1.0	12.7 ± 1.6	12.7 ± 0.7	1.0	13.3 ± 1.1	15.3 ± 1.6	**0.019**	13.2 ± 1.3	13.2 ± 1.2** §**	1.0	12.7 ± 0.8	16.1 ± 2.0	***0.003***	13.3 ± 1.0	15.0 ± 3.1	0.438	12.5 ± 0.8	16.9 ± 3.0	***1.3 × 10^−4^***	12.8 ± 1.4	14.3 ± 2.0** §**	0.653
QRSp (ms)	15.1 ± 1.5	16.8 ± 2.9	0.761	18.1 ± 2.2	18.2 ± 1.7	1.0	17.6 ± 4.3	22.6 ± 1.7	**0.005**	17.8 ± 3.2	18.4 ± 1.5** §**	1.0	16.1 ± 1.9	24.5 ± 2.3	***2.6 × 10^−5^***	17.2 ± 1.8	23.0 ± 5.5	***0.010***	15.4 ± 1.2	24.9 ± 3.1	***3.0 × 10^−9^***	17.9 ± 3.0	20.7 ± 2.3** §**	0.121
Q_peak_–S_peak_ (ms)	6.6 ± 0.6	5.9 ± 0.9	1.0	7.2 ± 1.3	5.9 ± 1.1	0.087	6.9 ± 1.1	5.9 ± 1.0	0.233	7.1 ± 0.9	6.3 ± 0.9	0.502	6.6 ± 0.6	5.7 ± 1.1	0.357	6.6 ± 1.2	5.9 ± 1.0	0.726	6.9 ± 0.9	6.3 ± 1.6	1.0	7.5 ± 0.7	6.0 ± 1.2	***0.039***
Q_peak_–S_end_ (ms)	8.8 ± 0.5	8.2 ± 1.2	1.0	9.3 ± 1.4	8.3 ± 1.0	0.286	9.5 ± 1.0	9.0 ± 1.5	1.0	9.2 ± 0.7	8.6 ± 0.5	1.0	8.8 ± 0.6	9.3 ± 2.4	1.0	9.9 ± 1.2	8.7 ± 1.3	0.859	8.7 ± 1.0	10.7 ± 2.9	0.123	9.1 ± 0.8	9.1 ± 2.1	1.0
QT_peak_ Interval (ms)	26.3 ± 2.3	29.0 ± 4.3	0.409	28.7 ± 3.1	29.0 ± 1.5	1.0	31.1 ± 7.4	37.2 ± 2.7	**0.048**	29.4 ± 4.2	29.3 ± 2.0** §**	1.0	28.1 ± 1.6	38.7 ± 3.3	***2.5 × 10^−5^***	29.3 ± 2.4	35.9 ± 7.7	***0.017***	26.6 ± 2.2	39.8 ± 5.4	***1.9 × 10^−7^***	27.7 ± 4.1	34.1 ± 3.8** §**	***0.013***
QT_peak_c Interval (ms)	29.5 ± 2.5	28.9 ± 2.1	1.0	30.8 ± 2.3	30.4 ± 2.4	1.0	32.0 ± 6.2	30.4 ± 3.8	1.0	30.7 ± 2.5	30.2 ± 2.2	1.0	30.0 ± 2.1	32.1 ± 3.9	1.0	31.1 ± 2.3	31.9 ± 6.1	1.0	30.1 ± 1.6	38.4 ± 5.9	***6.8 × 10^−5^***	31.7 ± 2.8	31.6 ± 1.9** §**	1.0
QT Interval (ms)	39.5 ± 2.7	43.3 ± 5.9	0.305	40.2 ± 3.2	40.3 ± 2.7	1.0	46.4 ± 8.1	59.2 ± 6.7	***0.002***	43.2 ± 7.1	42.5 ± 3.5** §**	1.0	43.1 ± 3.8	62.5 ± 6.6	***6.7 × 10^−9^***	41.9 ± 3.3	52.6 ± 6.4** §**	***7.7 × 10^−4^***	43.3 ± 4.7	63.4 ± 10.3	***4.7 × 10^−6^***	40.6 ± 4.8	51.8 ± 7.2** §**	***0.017***
QTc Interval (ms)	45.0 ± 2.1	43.2 ± 3.1	0.772	43.9 ± 2.1	42.7 ± 2.8	1.0	48.1 ± 6.2	47.2 ± 7.8	1.0	45.4 ± 3.9	44.1 ± 5.7	1.0	46.5 ± 3.1	50.9 ± 8.0	0.471	45.1 ± 2.6	45.5 ± 5.7	1.0	49.5 ± 1.7	60.8 ± 7.5	***4.9 × 10^−5^***	47.6 ± 2.5	47.4 ± 4.6** §**	1.0
R–S_peak_ (ms)	2.5 ± 0.3	2.6 ± 0.5	1.0	2.7 ± 0.4	2.5 ± 0.3	1.0	2.9 ± 0.3	2.5 ± 0.4	0.088	2.6 ± 0.4	2.5 ± 0.2	1.0	2.4 ± 0.5	2.3 ± 0.4	1.0	2.7 ± 0.5	2.6 ± 0.6	1.0	2.9 ± 0.5	2.5 ± 0.5	0.361	2.9 ± 0.5	2.5 ± 0.4	0.568
R–S_end_ (ms)	4.6 ± 0.6	4.9 ± 0.6	1.0	4.9 ± 0.8	4.8 ± 0.3	1.0	5.3 ± 0.3	5.6 ± 1.3	1.0	4.7 ± 0.6	4.8 ± 0.8	1.0	4.6 ± 0.8	5.9 ± 2.1	0.241	5.8 ± 0.8	5.5 ± 0.9	1.0	4.7 ± 0.7	6.9 ± 2.4	***0.010***	4.5 ± 0.6	5.7 ± 1.6	0.504
R–J (ms)	6.0 ± 0.4	6.5 ± 0.7	0.777	6.5 ± 1.0	6.5 ± 0.6	1.0	6.8 ± 0.8	8.7 ± 1.3	***5.3 × 10^−4^***	6.4 ± 0.6	6.7 ± 0.8** §**	1.0	5.9 ± 0.8	10.0 ± 1.5	***1.6 × 10^−7^***	7.0 ± 1.0	8.5 ± 1.6	0.104	6.0 ± 0.5	10.7 ± 2.7	***3.0 × 10^−7^***	6.4 ± 0.5	8.2 ± 1.1** §**	0.081
R–p (ms)	8.5 ± 1.0	11.1 ± 2.3	***0.036***	11.9 ± 1.9 **#**	12.0 ± 1.5	1.0	11.0 ± 4.1	15.9 ± 1.2	***0.002***	11.2 ± 2.7	11.9 ± 1.5** §**	1.0	9.2 ± 1.7	18.4 ± 1.6	***3.9 × 10^−8^***	10.7 ± 1.8	16.5 ± 4.1	***4.3 × 10^−4^***	8.8 ± 1.2	18.8 ± 3.1	***4.7 × 10^−10^***	11.5 ± 2.5	14.6 ± 2.6** §**	***0.047***
R–T_peak_ (ms)	20.1 ± 1.7	23.4 ± 3.6	0.051	22.8 ± 2.6	22.8 ± 1.3	1.0	24.5 ± 7.4	30.6 ± 3.0	***0.043***	22.5 ± 3.7	22.7 ± 1.8** §**	1.0	21.3 ± 1.4	32.6 ± 2.7	***1.9 × 10^−7^***	23.0 ± 2.1	29.3 ± 6.2	***0.003***	20.1 ± 2.0	33.8 ± 5.2	***1.8 × 10^−8^***	21.3 ± 3.4	27.9 ± 3.6** §**	***0.005***
R–T_peak_ c (ms)	23.0 ± 2.0	23.3 ± 1.3	1.0	24.7 ± 1.9	24.1 ± 2.3	1.0	25.4 ± 6.2	24.2 ± 4.1	1.0	23.7 ± 2.3	23.6 ± 2.1	1.0	23.2 ± 2.0	26.4 ± 4.0	0.320	24.7 ± 1.9	25.6 ± 5.3	1.0	23.4 ± 1.7	32.4 ± 5.9	***1.8 × 10^−5^***	25.0 ± 2.4	25.5 ± 2.3** §**	1.0
S_peak_–J (ms)	3.5 ± 0.4	3.8 ± 0.9	1.0	3.8 ± 0.8	4.0 ± 0.6	1.0	3.9 ± 0.6	6.2 ± 1.3	***5.2 × 10^−5^***	3.8 ± 0.7	4.2 ± 1.0** §**	1.0	3.5 ± 0.8	7.7 ± 1.4	***2.8 × 10^−7^***	4.3 ± 1.2	5.9 ± 1.8** §**	0.086	3.1 ± 0.5	8.2 ± 2.8	***1.2 × 10^−7^***	3.5 ± 0.5	5.7 ± 1.4** §**	***0.028***
S_peak_–p (ms)	5.9 ± 1.1	8.5 ± 2.3	***0.046***	9.1 ± 1.9 **#**	9.5 ± 1.4	1.0	8.1 ± 3.9	13.5 ± 1.3	***4.9 × 10^−4^***	8.6 ± 2.7	9.4 ± 1.3** §**	1.0	6.8 ± 1.9	16.1 ± 1.7	***4.4 × 10^−8^***	7.8 ± 1.7	13.9 ± 4.1	***3.7 × 10^−4^***	5.9 ± 1.4	16.3 ± 3.2	***3.8 × 10^−10^***	8.6 ± 2.6	12.1 ± 2.6** §**	***0.025***
S_peak_–T_peak_ (ms)	17.6 ± 1.6	20.8 ± 3.7	0.071	20.1 ± 2.4	20.3 ± 1.4	1.0	21.6 ± 7.2	28.1 ± 2.8	***0.022***	19.9 ± 3.6	20.3 ± 1.8** §**	1.0	18.9 ± 1.6	30.3 ± 2.9	***2.0 × 10^−7^***	20.3 ± 1.9	26.7 ± 6.2	***0.003***	17.2 ± 2.2	31.4 ± 5.2	***8.4 × 10^−9^***	18.3 ± 3.1	25.4 ± 3.7** §**	***0.002***
S_peak_–T_peak_ c (ms)	20.6 ± 1.9	20.7 ± 1.4	1.0	22.1 ± 1.8	21.6 ± 2.4	1.0	22.6 ± 6.0	21.6 ± 4.1	1.0	21.1 ± 2.3	21.2 ± 2.0	1.0	20.8 ± 2.1	24.0 ± 4.2	0.360	22.0 ± 2.1	22.9 ± 5.4	1.0	20.5 ± 2.1	30.0 ± 6.1	***1.7 × 10^−5^***	22.1 ± 2.1	23.0 ± 2.5** §**	1.0
ST Interval (ms)	30.8 ± 2.3	35.1 ± 5.8	0.117	31.6 ± 2.1	31.6 ± 2.8	1.0	37.0 ± 7.8	50.1 ± 6.7	***8.0 × 10^−4^***	33.7 ± 6.3	33.5 ± 3.2** §**	1.0	33.9 ± 3.7	54.1 ± 6.2	***2.2 × 10^−10^***	32.9 ± 2.6	43.4 ± 5.4** §**	***2.2 × 10^−4^***	33.9 ± 4.4	54.9 ± 10.0	***5.5 × 10^−7^***	31.2 ± 3.8	43.1 ± 6.6** §**	***0.005***
STc Interval (ms)	36.2 ± 1.6	34.9 ± 3.1	1.0	35.2 ± 1.1	34.0 ± 2.9	1.0	38.6 ± 5.9	38.4 ± 8.0	1.0	35.8 ± 3.3	35.1 ± 5.5	1.0	37.2 ± 3.1	42.7 ± 8.2	0.204	36.0 ± 2.2	36.6 ± 6.3	1.0	39.9 ± 1.9	52.5 ± 7.8	***1.0 × 10^−5^***	38.0 ± 1.9	38.8 ± 4.4** §**	1.0
S_end_–J (ms)	1.2 ± 0.4	1.6 ± 0.6	1.0	1.7 ± 0.4	1.7 ± 0.4	1.0	1.5 ± 0.8	3.1 ± 1.1	***5.6 × 10^−4^***	1.6 ± 0.6	1.9 ± 0.3** §**	1.0	1.1 ± 0.3	4.1 ± 1.4	***7.1 × 10^−6^***	1.1 ± 0.4	3.0 ± 1.4	***0.006***	1.4 ± 0.6	3.8 ± 1.8	***1.0 × 10^−4^***	1.9 ± 0.6	2.5 ± 0.9	1.0
S_end_–p (ms)	3.9 ± 1.5	6.2 ± 2.4	0.166	7.0 ± 2.2 **#**	7.2 ± 1.6	1.0	5.7 ± 4.0	10.4 ± 2.0	***0.010***	6.4 ± 3.1	7.1 ± 1.8	1.0	4.6 ± 2.2	12.5 ± 2.9	***2.4 × 10^−5^***	4.9 ± 2.5	11.0 ± 4.2	***0.002***	4.2 ± 1.6	11.8 ± 3.5	***1.3 × 10^−5^***	7.0 ± 2.8	8.9 ± 3.7	1.0
J–p (ms)	2.6 ± 1.2	4.7 ± 2.2	0.303	5.3 ± 2.4	5.5 ± 1.8	1.0	4.2 ± 3.4	7.2 ± 1.7	0.110	4.9 ± 2.8	5.2 ± 1.9	1.0	3.5 ± 1.9	8.4 ± 2.2	***6.1 × 10^−4^***	3.8 ± 2.3	8.0 ± 3.2	***0.009***	2.8 ± 1.2	8.1 ± 2.5	***3.2 × 10^−4^***	5.1 ± 2.6	6.4 ± 3.4	1.0
J–T_peak_ (ms)	14.1 ± 1.7	16.9 ± 3.5	0.165	16.0 ± 3.3	16.2 ± 1.5	1.0	17.8 ± 6.8	21.9 ± 3.5	0.336	16.0 ± 3.8	15.8 ± 2.0	1.0	15.4 ± 1.8	22.6 ± 3.4	***2.4 × 10^−4^***	16.0 ± 2.6	20.8 ± 5.1	***0.029***	14.1 ± 2.1	23.3 ± 3.8	***4.0 × 10^−6^***	14.7 ± 3.2	19.5 ± 3.8	***0.026***
J–T_end_ (ms)	27.3 ± 2.2	31.2 ± 5.4	0.132	27.5 ± 2.4	27.6 ± 2.3	1.0	33.1 ± 7.3	43.8 ± 7.6	***0.010***	29.8 ± 6.5	29.0 ± 3.5** §**	1.0	30.4 ± 4.0	46.4 ± 7.2	***2.0 × 10^−7^***	28.6 ± 3.3	37.6 ± 4.5** §**	***0.004***	30.8 ± 4.4	46.9 ± 9.0	***3.3 × 10^−5^***	27.6 ± 3.9	37.2 ± 7.0** §**	***0.026***
J–T_end_c (ms)	31.8 ± 1.7	31.1 ± 3.1	1.0	30.5 ± 1.4	29.6 ± 2.7	1.0	34.5 ± 5.6	34.0 ± 8.6	1.0	31.6 ± 4.0	30.3 ± 5.6	1.0	33.2 ± 3.3	36.8 ± 8.9	0.973	31.2 ± 2.1	31.8 ± 5.4	1.0	35.9 ± 2.1	44.8 ± 6.4	***6.5 × 10^−4^***	33.4 ± 2.2	33.6 ± 5.1** §**	1.0
T_peak_–T_end_ (ms)	13.2 ± 2.0	14.3 ± 3.7	1.0	11.5 ± 2.1	11.3 ± 2.1	1.0	15.3 ± 3.4	22.0 ± 5.0	***0.006***	13.8 ± 3.5	13.2 ± 2.5** §**	1.0	15.0 ± 3.5	23.8 ± 7.2	***0.002***	12.6 ± 3.2	16.7 ± 4.5** §**	0.518	16.7 ± 3.3	23.6 ± 6.4	***0.014***	12.9 ± 2.4	17.7 ± 4.8	0.226

The background color of the columns is grey for the Control groups and blue for the Adenine group. Heart rate (HR) was derived from either RR interval or PP interval durations. The c represents the parameter being corrected for heart rate. Results are presented as mean ± standard deviation. Two-way ANOVA plus Bonferroni’s multiple comparisons correction was used to detect significance between groups at each time point, considering regimen type and sex, and shown with a navy-blue *p*-value. If a sex-dependent interaction exists (*p* < 0.05) at each time point, it is depicted as # (black) to the Control group and § (blue) in the Adenine group. Abbreviations used: ms, milliseconds; bpm, beats per minute; c, corrected for heart rate.

## Data Availability

Data available upon request.

## Supplementary Materials

The following supporting information can be downloaded at: https://www.mdpi.com/article/10.3390/jcdd11110362/s1, Table S1: Comparison of cardiac body weight to cardiac morphological structures; Table S2: Cardiac morphological and structural characteristics; Table S3: Correlation of ECG parameters to heart rate; Table S4: Correlation of corrected ECG parameters to heart rate; Figure S1: Morphological and echocardiographic evaluation of left ventricular (LV) remodeling; Figure S2: Echocardiographic evaluation of left ventricular (LV) systolic function; Figure S3: Echocardiographic evaluation of left ventricular (LV) diastolic function; Figure S4: Echocardiographic evaluation of overall left ventricular (LV) function; Figure S5: Representative electrocardiogram (ECG) signals and changes in ECG parameters due to regimen type and duration.
