# Supplementary material for: Sex-Specific Changes in Cardiac Function and Electrophysiology During Progression of Adenine-Induced Chronic Kidney Disease in Mice
Source: J Cardiovasc Dev Dis. 2024 Nov 7;11(11):362. doi: 10.3390/jcdd11110362 (PMC11594452; doi:10.3390/jcdd11110362)
Supplement: Supplementary file 1 [file jcdd-11-00362-s001.zip › jcdd-3188047-supplementary.pdf]

Sex-Specific Changes in Cardiac Function and Electrophysiology During Progression of Adenine-Induced Chronic Kidney Disease in Mice

Dargam V. et al.

SUPPLEMENTARY TABLES AND FIGURES

***Supplementary Table S1.* Comparison of cardiac body weight to cardiac morphological structures.** Correlation coefficient measuring the linear correlation between R-R interval duration to the heart rate corrected ECG parameters.

Parameter	Correlation Coefficient			
	Control		Adenine	
	Body Weight (g)	Heart Weight (mg)	Body Weight (g)	Heart Weight (mg)
Body Weight (g)	1.0000	0.6609	1.0000	0.2051
Heart Weight (mg)	0.6609	1.0000	0.2051	1.0000
Atria Weight (mg)	0.2083	0.5863	0.2037	0.6571
Left Ventricle Weight (mg)	0.7062	0.8282	0.1049	0.9077
Right Ventricle Weight (mg)	0.3966	0.7656	0.2576	0.6251
Tibia Length (mm)	0.2686	0.154	-0.0487	0.1632

Supplementary Table S2. **Cardiac morphological and structural characteristics.** Body weight and cardiac structural characteristics of mice fed either a **Control (Healthy)** or **Adenine** (**CKD**) diet for up to 12 weeks.

	Week 3						Week 6						Week 9						Week 12					
	Male			Female			Male			Female			Male			Female			Male			Female		
	Control	Adenine		Control	Adenine		Control	Adenine		Control	Adenine		Control	Adenine		Control	Adenine		Control	Adenine		Control	Adenine	
	n=10M	n=10M	p-value	n=10F	n=10F	p-value	n=10M	n=10M	p-value	n=10F	n=10F		n=10M	n=10M	p-value	n=10F	n=10F	p-value	n=10M	n=10M	p-value	n=10F	n=10F	p-value
Body Weight (g)	25.8±1.6	18.9±1.1	8.6e-12	19.2±1.1#	18.6±2.0	1.0	27.8±2.2	17.3±1.3	3.6e-16	20.9±1.5#	18.5±1.1	0.007	31.1±2.5	15.7±1.0	2.0e-20	21.1±1.5#	17.2±1.4	6.7e-5	33.2±3.6	14.8±1.7	3.8e-19	22.3±1.3#	16.4±1.5	5.5e-6
Heart Weight (mg)	129.9±15.9	104.7±9.4	2.4e-4	109.9±11.4#	99.6±10.4	0.388	122.5±7.5	80.7±4.7	2.4e-12	106.7±8.5#	99.8±11.6§	0.459	127.1±9.4	90.2±12.0	7.5e-9	109.2±6.1#	84.5±12.1	2.5e-5	132.9±13.1	107.8±8.8	1.3e-4	115.2±9.9#	99.2±13.5	0.023
Atria Weight (mg)	10.4±3.3	7.8±1.8	0.066	7.7±1.6	6.8±1.6	1.0	8.5±2.5	5.1±1.1	0.037	8.5±3.9	7.4±1.8	1.0	9.2±3.3	6.2±2.3	0.083	8.1±1.8	5.5±2.7	0.182	9.0±1.9	7.9±1.6	1.0	8.0±2.1	6.8±1.7	0.941
LV Weight (mg)	85.8±9.2	74.8±6.6	0.014	78.2±5.6	71.8±8.0	0.366	87.2±6.7	57.9±4.0	3.0e-12	76.2±5.5#	69.3±7.2§	0.081	89.2±3.8	64.1±7.1	2.3e-10	77.8±4.7#	61.4±7.6	3.0e-6	93.3±8.7	79.0±8.0	0.001	81.7±5.2#	72.6±8.9	0.078
RV Weight (mg)	33.8±6.8	22.2±5.0	7.8e-4	24.0±7.5#	21.0±4.3	1.0	26.9±3.8	17.7±2.4	8.7e-6	22.0±4.0#	23.1±3.8§	1.0	28.8±4.5	19.9±5.1	6.9e-4	23.3±5.0	17.7±3.7	0.055	30.6±4.3	20.8±4.0	3.8e-4	25.5±6.0	19.9±4.7	0.082
Tibia Length (mm)	17.1±0.7	17.2±0.2	1.0	17.1±0.9	17.2±0.4	1.0	17.1±0.4	17.4±0.9	1.0	16.6±0.6	16.8±0.7	1.0	17.9±0.4	17.4±0.9	1.0	17.0±1.1	17.1±1.1	1.0	17.5±0.9	17.6±0.7	1.0	17.6±0.6	17.2±1.0	1.0
HW/BW (mg/g)	5.0±0.6	5.6±0.6	0.338	5.7±0.6	5.4±0.6	1.0	4.4±0.4	4.7±0.5	1.0	5.1±0.5#	5.4±0.5§	1.0	4.1±0.4	5.8±0.8	2.8e-6	5.2±0.5#	4.9±0.6§	1.0	4.0±0.3	7.3±0.8	2.5e-14	5.2±0.5#	6.0±0.6§	0.010
AW/BW (mg/g)	0.4±0.1	0.4±0.1	1.0	0.4±0.1	0.4±0.1	1.0	0.3±0.1	0.3±0.1	1.0	0.4±0.2	0.4±0.1	1.0	0.3±0.1	0.4±0.2	0.612	0.4±0.1	0.3±0.1	1.0	0.3±0.0	0.5±0.1	2.2e-7	0.4±0.1	0.4±0.1§	0.968
LVW/BW (mg/g)	3.3±0.3	4.0±0.4	0.002	4.1±0.2#	3.9±0.5	1.0	3.1±0.3	3.4±0.4	1.0	3.7±0.4#	3.8±0.3	1.0	2.9±0.3	4.1±0.5	1.0e-7	3.7±0.4#	3.6±0.3§	1.0	2.8±0.2	5.4±0.5	7.2e-17	3.7±0.3#	4.4±0.4§	2.8e-4
RVW/BW (mg/g)	1.3±0.3	1.2±0.3	1.0	1.2±0.4	1.1±0.2	1.0	1.0±0.2	1.0±0.1	1.0	1.1±0.2	1.2±0.2§	0.111	0.9±0.2	1.3±0.3	0.023	1.1±0.3	1.0±0.2	1.0	0.9±0.1	1.4±0.4	0.001	1.1±0.3	1.2±0.3	1.0
AW/HW (mg/mg)	0.1±0.0	0.1±0.0	1.0	0.1±0.0	0.1±0.0	1.0	0.1±0.0	0.1±0.0	1.0	0.1±0.0	0.1±0.0	1.0	0.1±0.0	0.1±0.0	1.0	0.1±0.0	0.1±0.0	1.0	0.1±0.0	0.1±0.0	1.0	0.1±0.0	0.1±0.0	1.0
LVW/HW (mg/mg)	0.7±0.0	0.7±0.0	0.046	0.7±0.1#	0.7±0.0	1.0	0.7±0.0	0.7±0.0	1.0	0.7±0.0	0.7±0.0	0.395	0.7±0.0	0.7±0.0	1.0	0.7±0.0	0.7±0.0	1.0	0.7±0.0	0.7±0.0	0.330	0.7±0.0	0.7±0.0	1.0
RVW/HW (mg/mg)	0.3±0.0	0.2±0.0	0.047	0.2±0.0	0.2±0.0	1.0	0.2±0.0	0.2±0.0	1.0	0.2±0.0	0.2±0.0	0.521	0.2±0.0	0.2±0.0	1.0	0.2±0.0	0.2±0.0	1.0	0.2±0.0	0.2±0.0	0.116	0.2±0.0	0.2±0.0	1.0
AW/TL (mg/mm)	0.6±0.2	0.5±0.1	0.064	0.5±0.1	0.4±0.1	1.0	0.5±0.1	0.3±0.1	0.034	0.5±0.2	0.4±0.1	1.0	0.6±0.2	0.4±0.1	0.052	0.5±0.1	0.3±0.1	0.120	0.5±0.1	0.5±0.1	1.0	0.5±0.1	0.4±0.1	1.0
LVW/TL (mg/mm)	5.0±0.5	4.3±0.4	0.003	4.6±0.3	4.2±0.4	0.190	5.1±0.3	3.3±0.2	2.9e-11	4.6±0.4#	4.1±0.5§	0.083	5.0±0.2	3.7±0.4	2.1e-8	4.6±0.3	3.6±0.3	4.3e-7	5.4±0.6	4.5±0.5	0.002	4.7±0.3#	4.2±0.5	0.281
RVW/TL (mg/mm)	2.0±0.4	1.3±0.3	8.9e-4	1.4±0.5#	1.2±0.3	1.0	1.6±0.2	1.0±0.2	5.5e-5	1.3±0.3	1.4±0.2§	1.0	1.6±0.3	1.1±0.3	0.038	1.4±0.3	1.0±0.2	0.063	1.8±0.3	1.2±0.2	4.7e-4	1.4±0.3	1.2±0.3	0.205

The background color of the columns is grey for the Control groups and blue for the Adenine group. Results are presented as mean ± standard deviation. Two-way ANOVA plus Bonferroni's multiple comparisons correction used to detect significance between groups at each time point, considering regimen type and sex. The p-value reported in this table depicts differences due to regimen type between corresponding sexes and regimens. If a sex-dependent interaction exists ($p<0.05$) at each time point, it is depicted **#** to the Control group and **§** in the Adenine group. Abbreviations used: **BW**, body weight; **HW**, heart weight; **LV**, left ventricle; **RV**, right ventricle; **AW**, atria weight; **LVW**, left ventricle weight; **RVW**, right ventricle weight; **TL**, tibia length.

***Supplementary Table S3*. Correlation of ECG parameters to heart rate.** Correlation coefficient measuring the linear correlation between R-R interval duration to duration of all other ECG parameters identified for analysis. The background color of the columns is grey for the Control groups and blue for the Adenine group.

Parameter	Correlation Coefficient		
	**Control**	**Adenine**	**Control + Adenine**
RR Interval	1.0000	1.0000	1.0000
PP Interval	0.9910	0.9798	0.9877
P-Wave	0.2339	0.1565	0.0556
PR Interval	0.4079	0.4523	0.4380
PR Segment	0.3550	0.4508	0.4680
Q_{start} - R	0.3836	0.4186	0.2507
Q_{start} - S_{peak}	0.4159	0.3885	0.1913
QRS Complex	0.4087	0.4498	0.4267
QRSJ	0.4368	0.5882	0.6515
QRSp	0.4247	0.5729	0.6785
Q_{peak} - S_{peak}	0.2590	0.1547	0.1132
Q_{peak} - S_{end}	0.2799	0.3539	0.2565
QT_{peak} Interval	0.6429	0.6218	***0.7392***
QT Interval	0.7293	0.6552	***0.7729***
R - S_{peak}	0.2393	0.0343	0.0572
R - S_{end}	0.2072	0.3241	0.3964
R - J	0.2480	0.5019	0.6297
R - p	0.3388	0.5028	0.6465
R - T_{peak}	0.6084	0.5802	***0.7242***
S_{peak} - J	0.1100	0.4769	0.6119
S_{peak} - p	0.2894	0.4916	0.6394
S_{peak} - T_{peak}	0.5983	0.5681	***0.7213***
ST Interval	0.7264	0.6197	***0.7601***
S_{peak} - J	0.0584	0.3808	0.5315
S_{end} -p	0.2493	0.3682	0.5346
J - p	0.5874	0.5123	0.6579
J - T_{peak}	0.5874	0.5123	0.6579
J - T_{end}	0.7050	0.5933	***0.7293***
T_{peak} - T_{end}	0.4049	0.5089	0.5944

Supplementary Table S4. **Correlation of corrected ECG parameters to heart rate.** Correlation coefficient measuring the linear correlation between R-R interval duration to the heart rate corrected ECG parameters. The background color of the columns is grey for the Control groups and blue for the Adenine group.

Parameter	Correlation Coefficient		
	**Control**	**Adenine**	**Control + Adenine**
$QT_{peak}c$ Interval	0.1701	0.1908	0.0
QTc Interval	0.0954	0.1298	0.0
R - T_{peak} c	0.1103	0.2411	0.0
S_{peak} - T_{peak} c	0.0741	0.2500	0.0
STc Interval	0.0126	0.1612	0.0

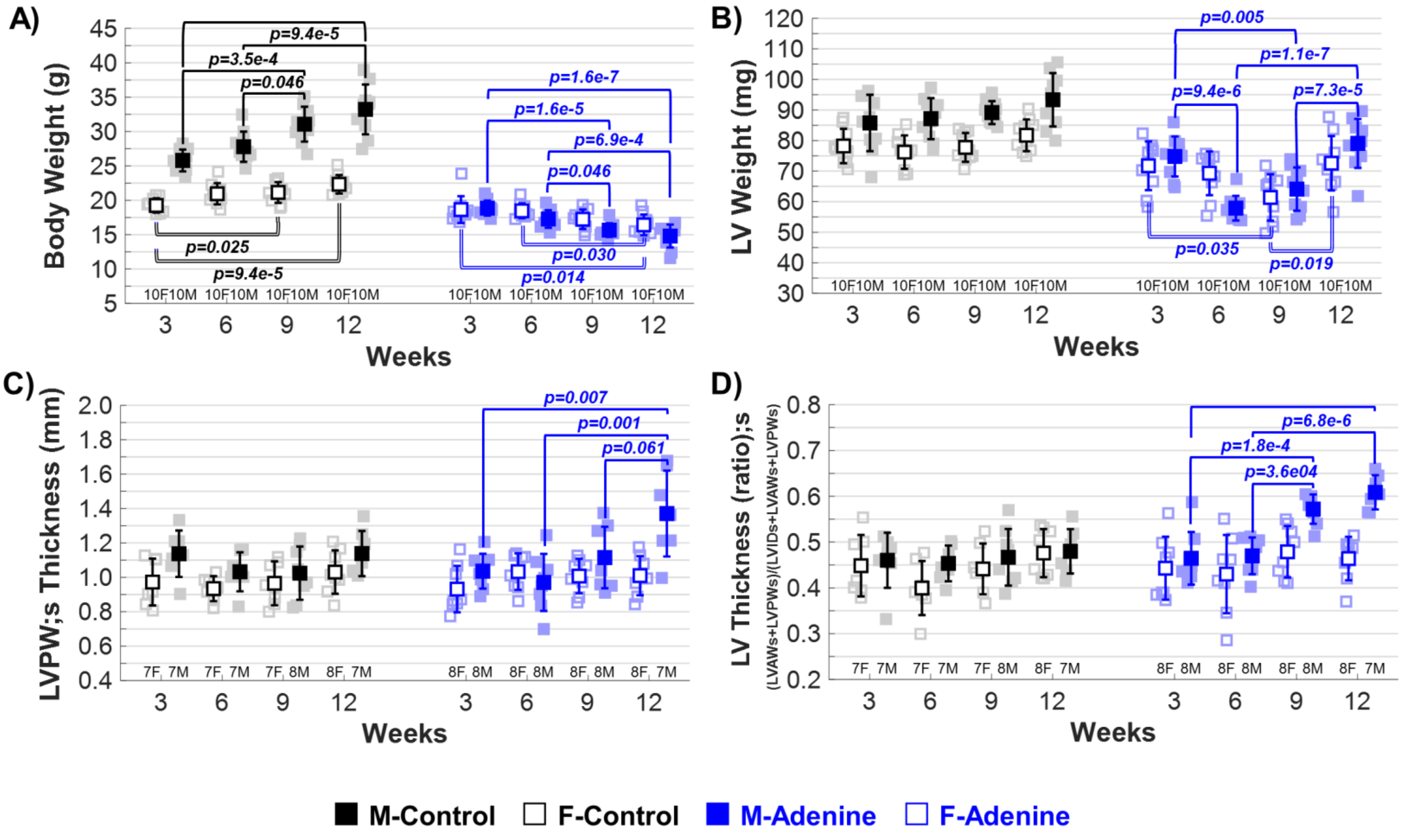

Supplementary Figure S1: Morphological and echocardiographic evaluation of left ventricular (LV) remodeling. Changes in ■ male and □ female mice throughout progression of either **control (Healthy)** or **adenine** (**CKD**) regimen**.** Differences based on regimen duration for the following: **A)** body weight, **B)** left ventricular (LV) weight, **C)** left ventricular posterior wall (LVPW), and **D)** LV thickness. Measures of LV wall thickness and inner diameter were calculated at systole (s). Results are presented as mean ± standard deviation. A one-way ANOVA (Bonferroni) was used to detect significance due to disease progression per sex and regimen type.

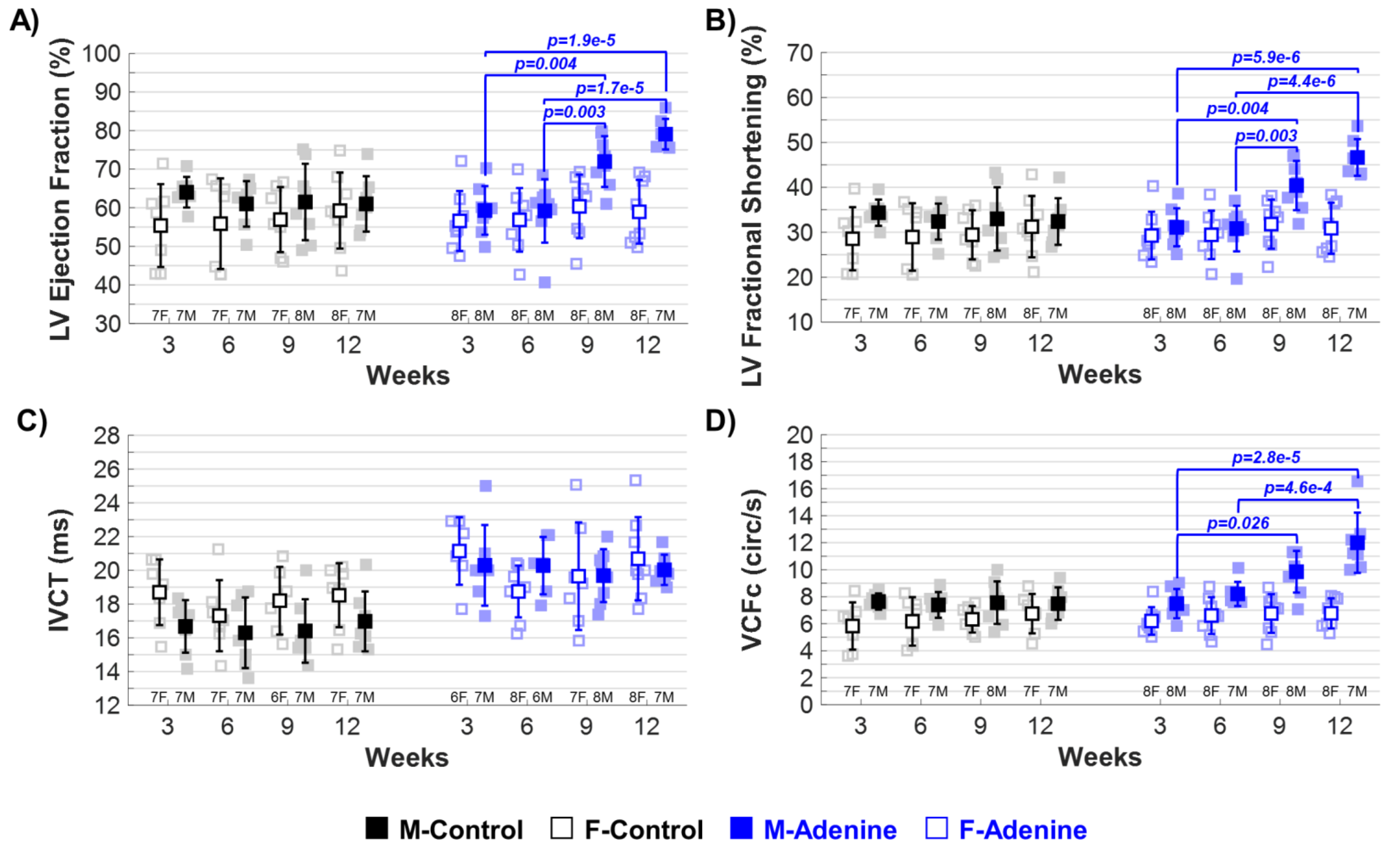

Supplementary Figure S2: Echocardiographic evaluation of left ventricular (LV) systolic function. Changes in ■ male and □ female mice throughout progression of either **control (Healthy)** or **adenine (CKD)** regimen. Echocardiogram-based parameters of LV systolic function tracked throughout disease progression included: **A)** LV ejection fraction, **B)** fractional shortening, **C)** isovolumetric contraction time (IVCT), and **D)** velocity of circumferential fiber shortening corrected for heart rate (VCFc). Results are presented as mean ± standard deviation. A one-way ANOVA (Bonferroni) was used to detect significance due to disease progression per sex and regimen type.

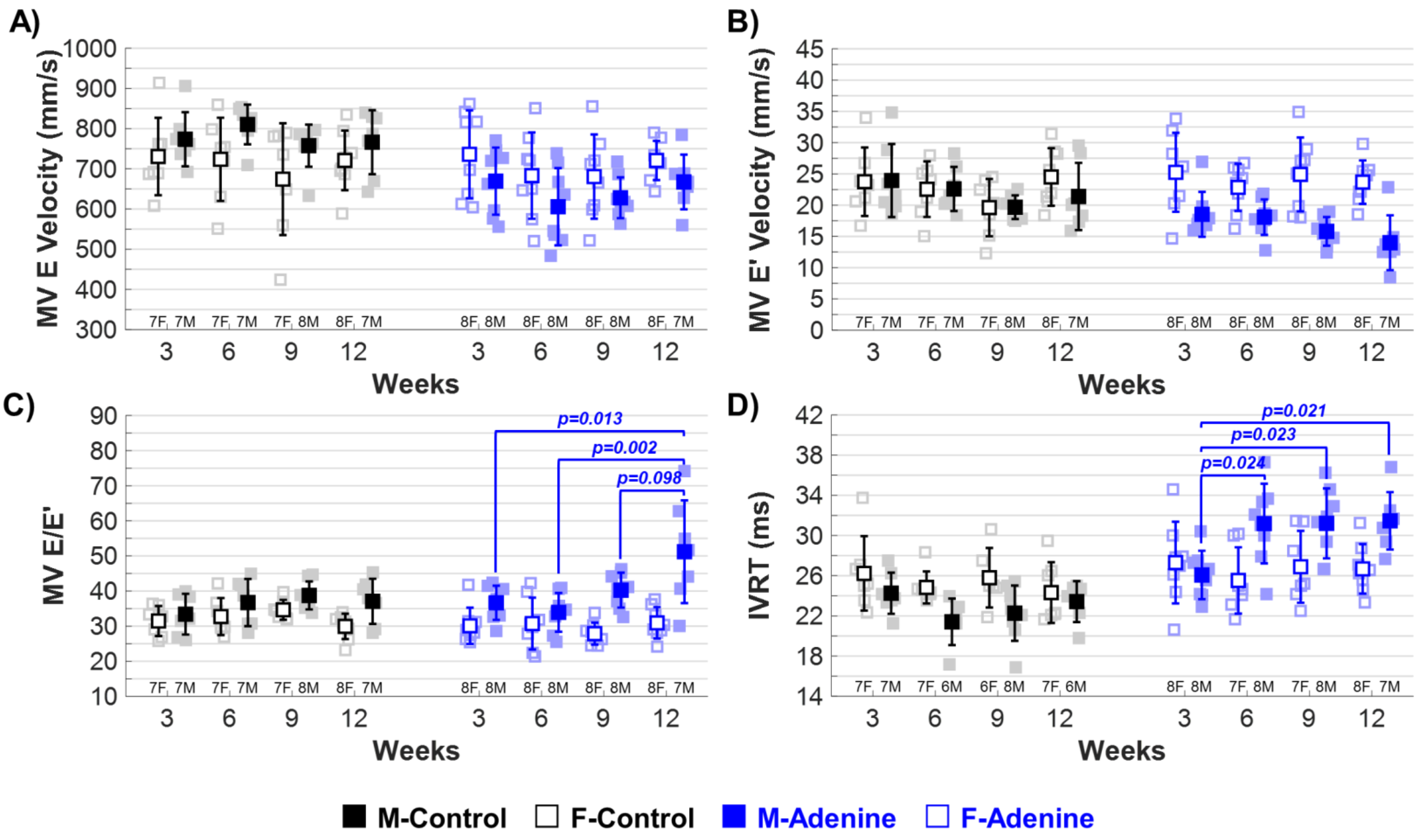

Supplementary Figure S3: Echocardiographic evaluation of left ventricular (LV) diastolic function. Changes in ■ male and □ female mice throughout progression of either **control (Healthy)** or **adenine (CKD)** diet regimen. Echocardiogram-based parameters of LV diastolic function tracked throughout disease progression included: **A)** mitral valve early flow velocity (MV E), **B)** mitral annulus velocity (MV E'), **C)** MV E/E', and **D)** isovolumetric relaxation time (IVRT). Results are presented as mean ± standard deviation. A one-way ANOVA (Bonferroni) was used to detect significance due to disease progression per sex and regimen type.

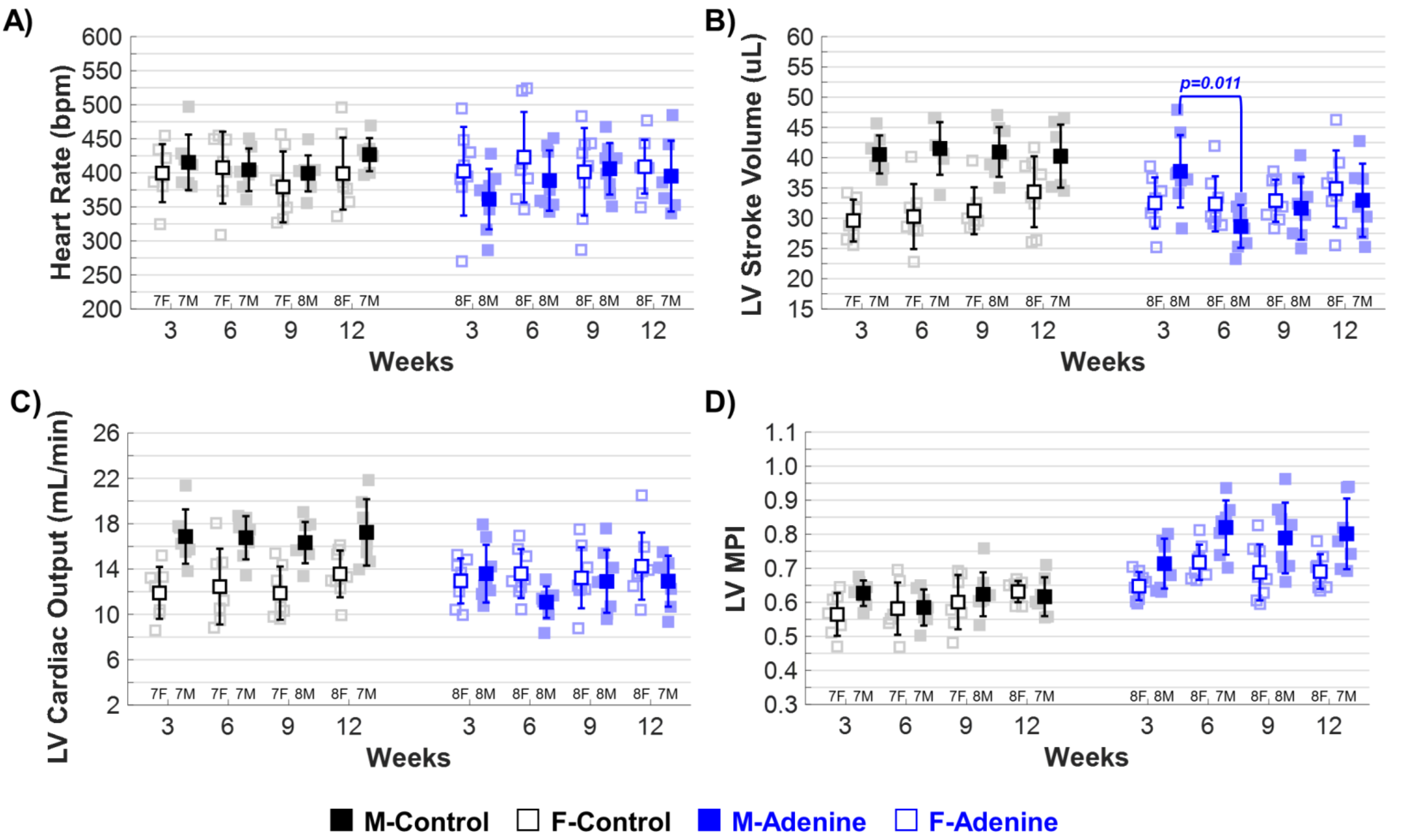

Supplementary Figure S4: Echocardiographic evaluation of overall left ventricular (LV) function. Changes in in ■ male and □ female mice throughout progression of either **control (Healthy)** or **adenine (CKD)** diet regimen. Echocardiogram-based parameters of cardiac function tracked throughout disease progression included: **A)** heart rate, **B)** LV stroke volume, **C)** LV cardiac output, and **D)** myocardial performance index (MPI). Results are presented as mean ± standard deviation. A one-way ANOVA (Bonferroni) was used to detect significance due to disease progression per sex and regimen type.

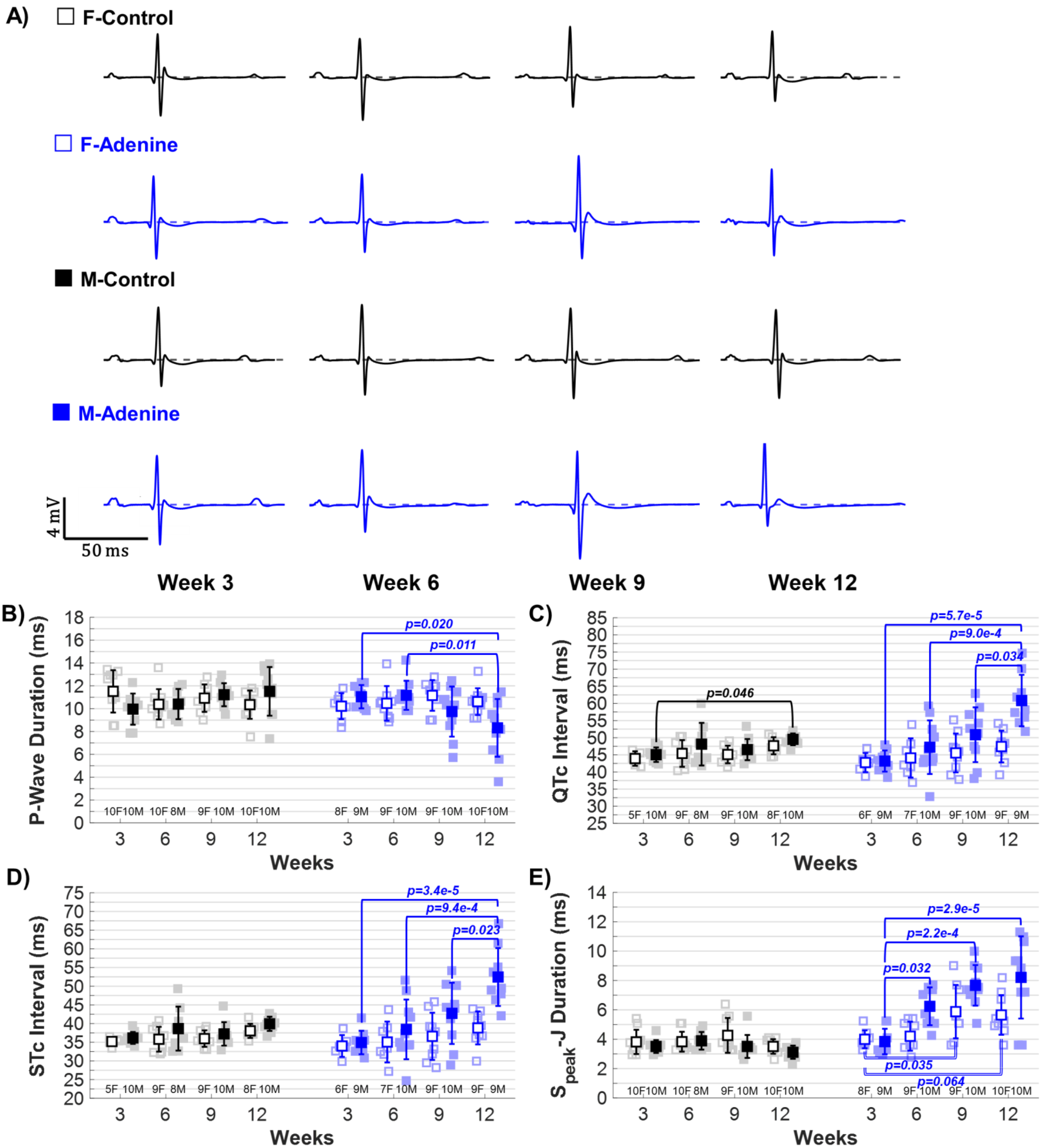

Supplementary Figure S5: Representative electrocardiogram (ECG) signals and changes in ECG parameters due to regimen type and duration. A) Cardiac electrophysiological changes in ■ male and □ female mice measured via ECG and quantified using Lead I throughout progression of either **control (Healthy)** or **adenine (CKD)** regimen. ECG-based parameters tracked throughout disease progression included **B)** P-wave duration, **C)** QTc interval duration, **D)** STc interval duration, and **E)** S_{peak}-J duration. Results are presented as mean ± standard deviation. A one-way ANOVA (Bonferroni) was used to detect significance due to disease progression per sex and regimen type.
